# Integrated single-cell analysis revealed immune dynamics during Ad5-nCoV immunization

**DOI:** 10.1038/s41421-021-00300-2

**Published:** 2021-08-10

**Authors:** Qiqi Cao, Shipo Wu, Chuanle Xiao, Shuzhen Chen, Xiangyang Chi, Xiuliang Cui, Hao Tang, Wenru Su, Yingfeng Zheng, Jiayong Zhong, Zhaomin Li, Fang Li, Haijia Chen, Lihua Hou, Hongyang Wang, Wen Wen

**Affiliations:** 1 International Cooperation Laboratory on Signal Transduction, Eastern Hepatobiliary Surgery Hospital, Second Military Medical University / Naval Medical University, Shanghai, China; 2 Beijing Institute of Biotechnology, Beijing, China; 3 State Key Laboratory of Ophthalmology, Zhongshan Ophthalmic Center, Sun Yat-sen University, Guangzhou, Guangdong China; 4 National Center for Liver Cancer, Second Military Medical University / Naval Medical University, Shanghai, China; 5 Department of Respiratory and Critical Care Medicine, Changzheng Hospital, Second Military Medical University / Naval Medical University, Shanghai, China; 6 Department of Critical Care, Wuhan Huoshenshan Hospital, Wuhan, Hubei China; 7 HuaAn McAb Biotech Company, Hangzhou, Zhejiang China; 8 Guangzhou SALIAI Stemcell Science and Technology Co., Ltd., Guangzhou, Guangdong China; 9 Fudan University Shanghai Cancer Center; Department of Oncology, Shanghai Medical College, Fudan University, Shanghai, China; 10 Department of Laboratory Medicine, Eastern Hepatobiliary Surgery Hospital, Second Military Medical University / Naval Medical University, Shanghai, China

**Keywords:** Bioinformatics, Immunology

## Abstract

Coronavirus disease 2019 (COVID-19), driven by SARS-CoV-2, is a severe infectious disease that has become a global health threat. Vaccines are among the most effective public health tools for combating COVID-19. Immune status is critical for evaluating the safety and response to the vaccine, however, the evolution of the immune response during immunization remains poorly understood. Single-cell RNA sequencing (scRNA-seq) represents a powerful tool for dissecting multicellular behavior and discovering therapeutic antibodies. Herein, by performing scRNA/V(D)J-seq on peripheral blood mononuclear cells from four COVID-19 vaccine trial participants longitudinally during immunization, we revealed enhanced cellular immunity with concerted and cell type-specific IFN responses as well as boosted humoral immunity with SARS-CoV-2-specific antibodies. Based on the CDR3 sequence and germline enrichment, we were able to identify several potential binding antibodies. We synthesized, expressed and tested 21 clones from the identified lineages. Among them, one monoclonal antibody (P3V6-1) exhibited relatively high affinity with the extracellular domain of Spike protein, which might be a promising therapeutic reagent for COVID-19. Overall, our findings provide insights for assessing vaccine through the novel scRNA/V(D)J-seq approach, which might facilitate the development of more potent, durable and safe prophylactic vaccines.

## Introduction

The coronavirus disease 2019 (COVID‑19) pandemic, caused by severe acute respiratory syndrome coronavirus 2 (SARS-CoV-2), affects 191 countries and territories. Common symptoms include fever, cough, fatigue, breathing difficulties, and loss of smell. Complications may include pneumonia and acute respiratory distress syndrome. As of June 20, 2021, more than 178 million confirmed cases and 3,857,719 deaths have been reported worldwide^1^. No validated therapeutics or specific antiviral medications are available for COVID-19. The impact on global health and the scale of socioeconomic damage is driving intense vaccine development, accelerated by multiple novel technology platforms. Genotypic and protein structural analysis of potent neutralizing antibodies from convalescent donors has shed some light on vaccine design^2–5^. Spike glycoprotein (S protein) is responsible for the initial binding of host cells through angiotensin-converting enzyme 2 (ACE2). Because of the binding and protruding nature of S protein and its receptor-binding domain (RBD), most investigators are now pursuing S protein or the RBD as vaccine targets.

At present, scientists are working on ~160 vaccines, with more than 60 of them on phase 2 or 3 trial in 41 countries. There are 8 authorized vaccines in early or limited use across several countries and 8 vaccines have been approved for full use so far (updated June 18, 2021)^6^. Clinical evaluation of the immune response is a critical step to support the approval of vaccines, which usually takes immunogenicity, safety and efficacy as basic considerations^7^. Generally, we rely heavily on antibody titers as proof of protection since it can be measured with minimal blood sample. However, antibodies, representation of humoral immune response, are not always sufficient for protection. Some studies provided evidence that effective prophylactic vaccines against replicating viruses should engage strong cellular T cell immunity^8,9^. The critical protection of T cell-eliciting vaccines against infections have not been clearly defined and probably understudied. With the aim of providing a safe and effective vaccine as early as possible, rapid and robust methods for vaccine evaluation are urgently needed.

Single-cell RNA sequencing (scRNA-seq) is an arising technique that enables transcriptome-wide gene expression measurement and a dynamic view of cell lineage at single-cell resolution. During the COVID-19 pandemic, this method facilitated the identification of the ACE2/TMPRSS2 expression distribution among different cell clusters in patients with COVID-19^10^. It also helped depict the immune landscape change during the course of COVID-19 from peripheral blood and bronchoalveolar lavage fluid^11,12^. Single-cell 5′ mRNA and V(D)J sequencing (scRNA/V(D)J-seq), is an unbiased method specialized for quantitating antigen receptor diversity^13^. It can be used for rapid discovery of large, diverse panels of high-affinity antigen-specific antibodies. A joint research team led by Sunney Xie identified antigen-binding clonotypes by high-throughput scRNA/V(D)J-seq, revealing neutralizing antibodies based on predicted CDR3 structures^14^. These findings showed that combined with certain strategies, this technique could help identify potent antibodies that could greatly assist in the intervention of prevailing and emerging pandemics, such as COVID-19^15^. Collectively, B and T cell clonality, vaccine-induced cell phenotypes, and transcriptional signatures are all important avenues of investigation that can be achieved through scRNA-seq.

Here, we used scRNA-seq as a tool for vaccine evaluation on peripheral blood mononuclear cells (PBMCs) from four participants who were engaged in a phase 1 trial of an Ad5-based recombinant vaccine (Ad5-nCoV, trade name: Convidecia™) in Wuhan, China. CanSino Biologics’ Convidecia (Ad5-nCoV) is a genetically engineered vaccine candidate with replication-defective adenovirus type 5 which encodes a full-length S protein of SARS-CoV-2. It was the first vaccine to enter a phase 1 trial with first-in-human data^16^. On February 25, 2021, Ad5-nCoV has been granted conditional marketing authorization by the National Medical Products Administration of China. Globally, it received authorization for emergency use in Mexico, Pakistan and Chile. We analyzed three timepoints, pre-vaccination (day 0) and days 14 and 28 after vaccination, and observed the dynamics in both cellular and humoral immune responses to evaluate the efficiency of Ad5-nCoV at a single-cell resolution. Furthermore, we screened for potential neutralizing antibodies with optimized strategies. In total, 21 neutralizing antibody candidates were selected by high-throughput scRNA/V(D)J-seq from the participants. We identified five monoclonal antibodies (mAbs), and the most potent mAb, P3V6-1, exhibited a medium effective concentration (EC_50_) of 0.02598 μg/mL against the extracellular domain (ECD) of the S protein (S-ECD). Overall, with timepoint sampling and tailored analysis, we presented an experimental methodology that used scRNA/V(D)J-seq to characterize the inter-timepoint, dosage-related immune landscape of clinical trial participants, and applied it to vaccine evaluation.

## Results

### Profiling of peripheral immune cells by scRNA-seq before and after Ad5-nCoV vaccination

**Fig. 1:**
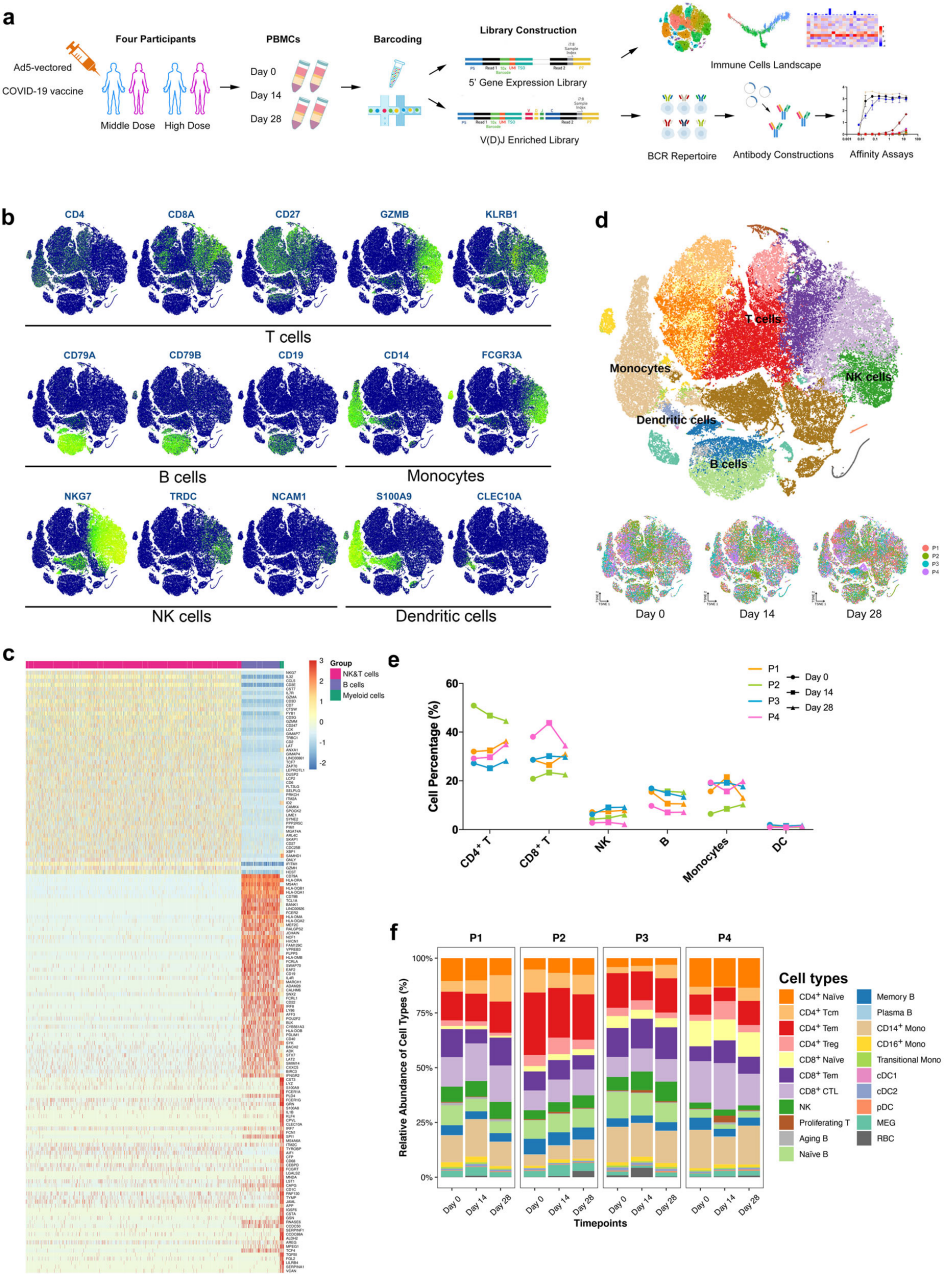
Dissection of immune dynamics during immunization with scRNA-seq. **a** Workflow of PBMCs collection, processing, sequencing, data analysis, and antibody validation. Cells from four participants at three timepoints were subjected to scRNA-seq and scV(D)J-seq. Antigen-specific antibodies were predicted and screened based on the CDR3 sequence from scBCR-seq. PBMCs, peripheral blood mononuclear cells. **b** *t*-SNE projection of canonical markers of each cell type. Cells are annotated based on differential expression analysis on orthogonally discovered clusters. **c** Heatmap revealing the scaled expression of DEGs for B cells, NK and T cells, and myeloid cells. **d** ScRNA-seq visualization with *t*-SNE analysis of PBMCs (*n* = 92,456) from all participants and timepoints sampled (top), and *t*-SNE annotated by timepoints and colored by individuals (bottom). **e** Proportions of major cell types from each participant at different timepoints assayed in this study. **f** Proportions of different cell types in the total PBMCs from participants at different timepoints by scRNA-seq.

After performing quality controls, we sequenced a total of 92,456 cells from four participants before the vaccination and on days 14 and 28 after the vaccination, with an average of 1306 detected genes per participant per timepoint (Supplementary Tables S1, S2). Two of the participants (P1 and P2) received a middle-dose intramuscular injection of the vaccine (1 × 10^11^ viral particles), while the other two (P3 and P4) received high-dose injections (1.5 × 10^11^ viral particles) (Fig. 1a). To assign cell identities, we analyzed an integrated cell-by-gene expression matrix and performed dimensional reduction by *t*-distributed stochastic neighbor embedding (*t*-SNE) and graph-based clustering, which yielded 30 clusters (Supplementary Fig. S1a). Most differentially expressed genes (DEGs) of each cluster and canonical lineage markers were both taken into consideration for manually annotated clusters.

We first separated total cells coarsely into several main cell types, including monocytes, dendritic cells (DCs), T cells, natural killer (NK) cells and B cells. Each cell type was then finely subdivided according to previously published transcriptional profiles (Fig. 1b, c). With this approach, we annotated eight T cell phenotypic subtypes, namely, naïve CD4^+^ T cells (CD4^+^CCR7^+^LEF1^+^TCF7^+^), central memory CD4^+^ T cells (CD4^+^ Tcms, CD4^+^CCR7^+^AQP3^+^CD69^+^), effector memory CD4^+^ T cells (CD4^+^ Tems, CD4^+^CCR6^+^CXCR6^+^CCL5^+^PRDM1^+^), regulatory T cells (CD4^+^ Tregs, CD4^+^FOXP3^+^), naive CD8^+^ T cells (CD8A^+^CCR7^+^LEF1^+^TCF7^+^), effector memory CD8^+^ T cells (CD8^+^ Tcms, CD8A^+^GZMK^+^), cytotoxic CD8^+^ lymphocytes (CD8^+^ CTLs, CD8A^+^GZMB^+^GNLY^+^PRF1^+^) and proliferating T cells (TYMS^+^ MKI67^+^); four B cell subtypes, namely, naive B cells (CD19^+^MS4A1^+^IGHD^+^ IGHM^+^IL4R^+^TCL1A^+^), memory B cells (CD27^+^CD38^+^IGHG^+^), plasma cells (XBP1^+^MZB1^+^) and aging B cells (TBX21^+^); and six myeloid cells, namely, classical monocytes (CD14^++^), nonclassical monocytes (CD16^++^CD14^−/+^), intermediate monocytes (CD14^++^CD16^+^), conventional DCs 1 (cDC1s, CLEC9A^+^), cDC2 (CD1C^+^) and plasmacytoid DCs (pDCs, CLEC4C^+^CD123^+^). Most clusters consisted of cells from every participant and timepoint (Fig. 1d), indicating that common immune traits, rather than technical artifacts, drove the variation among clusters.

We next quantified changes in the cell type proportions during vaccination (Supplementary Fig. S1b). As shown in Fig. 1e, the general patterns of the main PBMC populations were incomparable across timepoints, suggesting that Ad5-nCoV did not induce turbulence of immune cells (Fig. 1e, f). However, the proportion of CD4^+^ Tcms was increased significantly between day 14 and 28 (Supplementary Fig. S1c). The increased CD4^+^ Tcms may produce cytokines directly associated with better T cell secondary expansion, such as interleukin (IL)-2^17^. In addition, CD16^+^ monocytes and pDCs displayed expansions at day 14 and day 28, respectively, both of which have a great potential in producing cytokines^18^ (Supplementary Fig. S1c). PDCs, especially, are involved in the initiation of antiviral immune responses through their interaction with other innate and adaptive immune cells^19^. Having mapped cell type frequency dynamics, we were then able to generate a deep transcriptional map of the immune cell state.

### Humoral immune response and expanded BCR cloning are triggered by the Ad5-nCoV

**Fig. 2:**
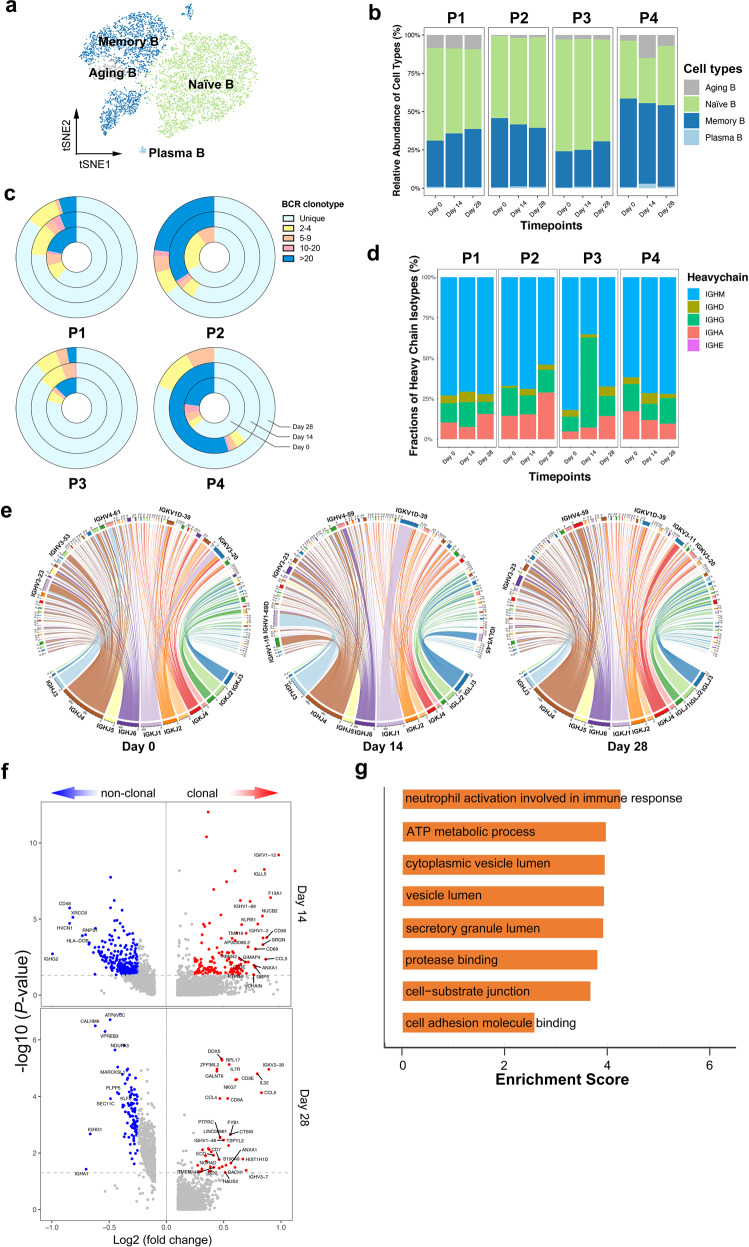
Humoral immune response and expanded BCR cloning during immunization. **a***t*-SNE analysis of B cells subtypes from all participants and timepoints. **b** Proportions of the B cell subtypes across participants and timepoints. **c** The pie plot showing the BCR clone differences across timepoints. The complexity and distribution of clonotypes differed among samples. **d** The bar plot showing the relative percentage of each isotype by each participant and timepoint. **e** The circus plots of the rearrangements of the BCR chains split by timepoints. The arc length of each segment corresponds to the relative frequency of each VDJ gene family. Within each plot, pairing VDJ gene segments are represented by colored ribbons. Ribbon width corresponds to frequency of the represented pairing. **f** The volcano plot showing the DEGs of expanded *vs.* non-expanded memory B cells and plasma cells at day 14 and day 28 post-vaccination. Genes with greatest fold changes and significant *P* values were annotated in the plot. **g** The enriched GO terms of the DEGs in expanded B cells upregulated 28 days post-vaccination.

By projecting the gene expression data of B cells in *t*-SNE map, we identified four B cell clusters, namely, naive B cells, memory B cells, plasma cells, and aging B cells (Fig. 2a; Supplementary Fig. S2a). Since there was no significant change in the proportion of B cells (Fig. 2b; Supplementary Fig. S2b), we used single-cell BCR sequencing (scBCR-seq) to assess the clonal BCR expansion status. Based on the timepoint, we found that each participant displayed different clonal patterns at different timepoints. P4 at day 14 after vaccination had significantly expanded clones compared to the status before vaccination (Fig. 2c). At the individual level, P3 experienced remarkable shifts in BCR isoforms compared with other participants (Fig. 2d). The diverse reactions of B cells and BCR isoforms suggested individual variation toward vaccination, which could be revealed by scBCR-seq, supporting the assumption that B cells preferentially experienced unique clonal V(D)J rearrangements when the subject received high-dose immunization. Moreover, quantification of the most highly expanded (maximum) clone for each timepoint showed that the maximum clones were higher at day 14 than at day 0, despite the expansion seemingly subsiding over time (day 28) (Supplementary Fig. S2c).

To characterize the diversity and preference in gene usage, we calculated the barcode frequency of the immunoglobulin heavy chain variable region (IGHV), immunoglobulin kappa chain variable region (IGKV) and immunoglobulin light chain variable region (IGLV). By gene usage analysis of the IG segments, we found that certain V(D)J genes such as IGHV3-23/IGHV4-59, IGKV1D-39/IGKV3-20, and IGHJ1/IGHJ4 were more frequently observed than other germlines. Besides, B cell lineages such as IGHV1-69D, IGKV1D-39, and IGLV5-45 were clonally expanded after vaccination (Fig. 2e; Supplementary Fig. S2d). Overall, most of the analyzed samples showed a polyclonal pattern of BCRs, exhibited the germline-gene preference, as previous findings indicated^20–23^. IGHV3-53 is the most frequently detected IGHV gene among SARS-CoV-2 RBD-targeting antibodies, which was not as highly upregulated as expected in our observation^24,25^. The possible explanation may be that not all the antibodies induced by Ad5-nCoV were SARS-CoV-2 RBD targeted.

Memory B cells and plasma cells play an important role in the development of adaptive immunity as they synergistically work and induce specific antibodies. To understand the functional status of expanded cloned B cells, we performed DEG analysis between the cloned B cells and uncloned B cells in the subset of memory B cells and plasma cells. As expected, there was increased expression of *S100A9*, *IGLL5*, *CD69*, *CD38,* and *CCL4*, illustrating the superior effector functions of the expanded cloned B cells (Fig. 2f). Gene ontology (GO) analysis revealed an increased metabolic process and vesicle lumen secretion in clonal expanded B cells, which was in accordance with the demanding needs of antibody synthesis and immune response activation (Fig. 2g).

### Antibody design and validation based on germline and CDR3 analysis

**Fig. 3:**
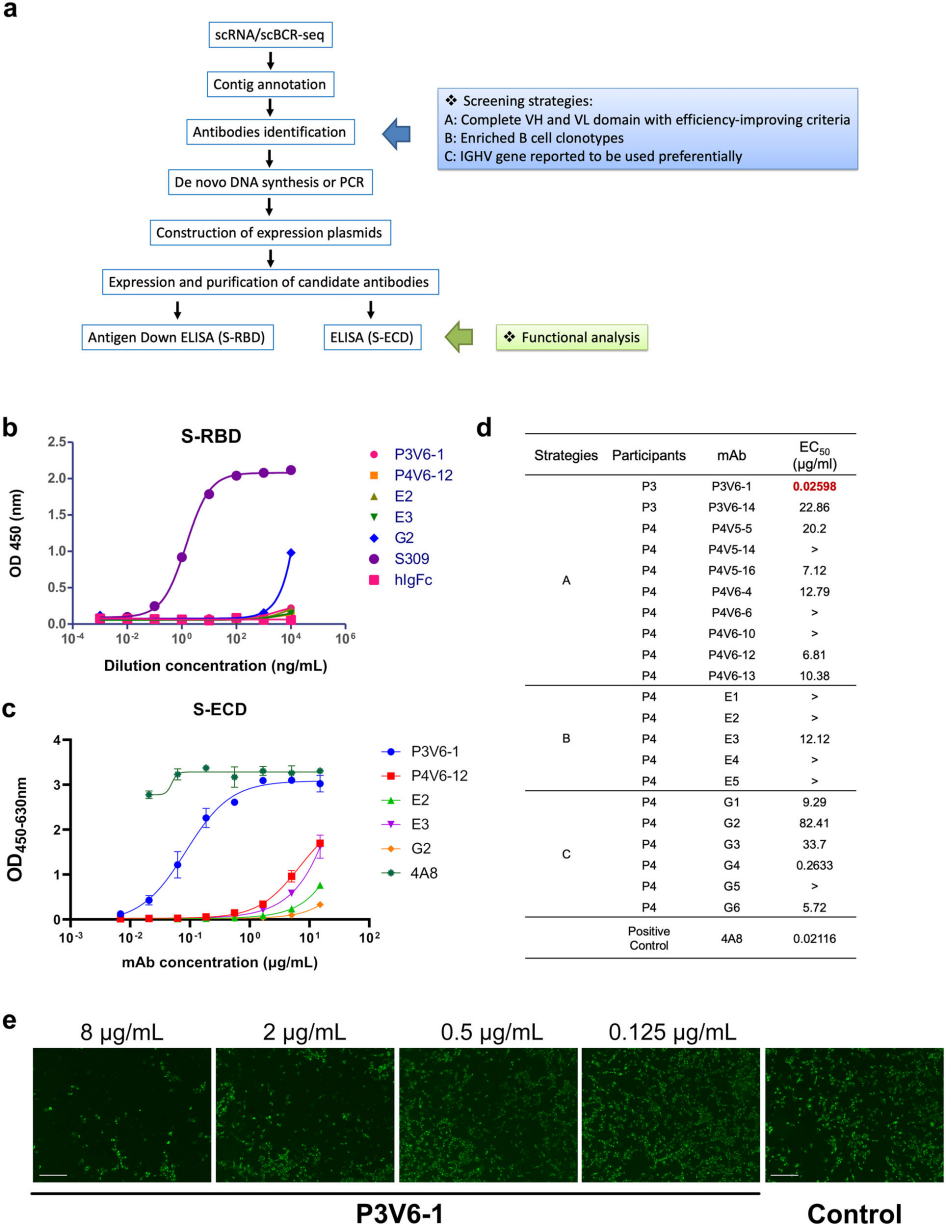
Antibody design and validation based on germline and CDR3 analysis. **a** Workflow of antibody identification and functional analysis. Potential antibody sequences were predicted according to screening strategies described in “Materials and methods”. The antibodies were then constructed and expressed for further functional analysis. **b** Binding curves of five representative mAbs to S-RBD. S309 is a positive control while hIgFC is a negative control. **c** Binding curves of five representative mAbs to S-ECD. 4A8 is a positive control that was reported to bind the S-ECD of SARS-CoV-2. **d** Diagram showing the binding of mAbs (EC_50_ value) to S-ECD determined by ELISA. EC_50_ values greater than 100 μg/mL are indicated as ">". **e** Pseudotyped virus neutralization assay to test the neutralization potency with gradient diluted P3V6-1. Infected cells were identified as GFP-positive cells. Images were obtained by using fluorescence microscopy. Scale bars, 40 µm.

Upon antigen stimulation, B cells proliferate and undergo clonal expansion. B cells expressing neutralizing IgA or IgG may be amplified by the immune system during immunization, which provide antigen specificity and cell ancestry. The germline preference information and CDR3 analysis provided a framework for the rational screening for SARS-CoV-2-specific antibodies. Guided by this rationale and the results of scBCR-seq, we utilized several strategies (see Fig. 3a and “Materials and methods”) to identify the sequence of antibodies. In total, 21 potential antibodies were expressed, purified, and tested for their binding properties for SARS-CoV-2 (for the sequences of light and heavy chains, see Supplementary Dataset S1).

The S protein is a large type I transmembrane protein containing two subunits, S1 and S2, both of which comprise an ECD and a single transmembrane helix. S1 consists of the N-terminal domain (NTD) and RBD^26^. To screen for S protein-specific antibodies, an RBD-mFc fusion protein was used to detect whether the antibody recognizes the RBD (for the sequence of RBD-mFc fusion protein, see Supplementary Dataset S2). The antigen-down enzyme-linked immunosorbent assay (ELISA) showed that only G2 had mild binding ability for Spike-RBD (S-RBD) (Fig. 3b; Supplementary Fig. S3a). Next, we performed ELISA by using 4A8, a published neutralization antibody binding to S-NTD, as a positive control to characterize antibodies that bind S-ECD^21^. Among these mAbs, five S-ECD-specific mAbs were identified, and P3V6-1 showed the most potent binding ability (Fig. 3c; Supplementary Fig. S3b). The NTD-specific antibodies were recognized on the basis of EC_50_ values (Fig. 3d). To test the neutralizing potency of P3V6-1, pseudotyped virus neutralization assay was applied to observe the decrease of GFP-positive cell number. After P3V6-1 treatment, the expression of viral protein decreased markedly in a dose-dependent manner (Fig. 3e).

Additionally, the clinical trial on Ad5-nCoV suggested that participants in the high-dose group produced elevated titers of neutralizing antibodies against S protein, RBD region and pseudovirus. Given the dose-dependent effect elicited by vaccination, we observed IgA/IgG counts and IgLC/IgKC ratio changes during immunization and validated consistent shifts in P3 and P4 (Supplementary Fig. S3c).

Overall, in line with the antibody titer results, Ad5-nCoV triggered the production of SARS-COV-2-specific antibodies with S-ECD-binding ability. One mAb, named P3V6-1, exhibits neutralization potency against pseudotyped SARS-CoV-2. Our results showed that the antigen-specific antibodies can be directly selected based on predicted CDR3 structures and germline information, which enlightens the antibody and vaccine design.

### The cellular immune response peaked at day 14 post-vaccination

**Fig. 4:**
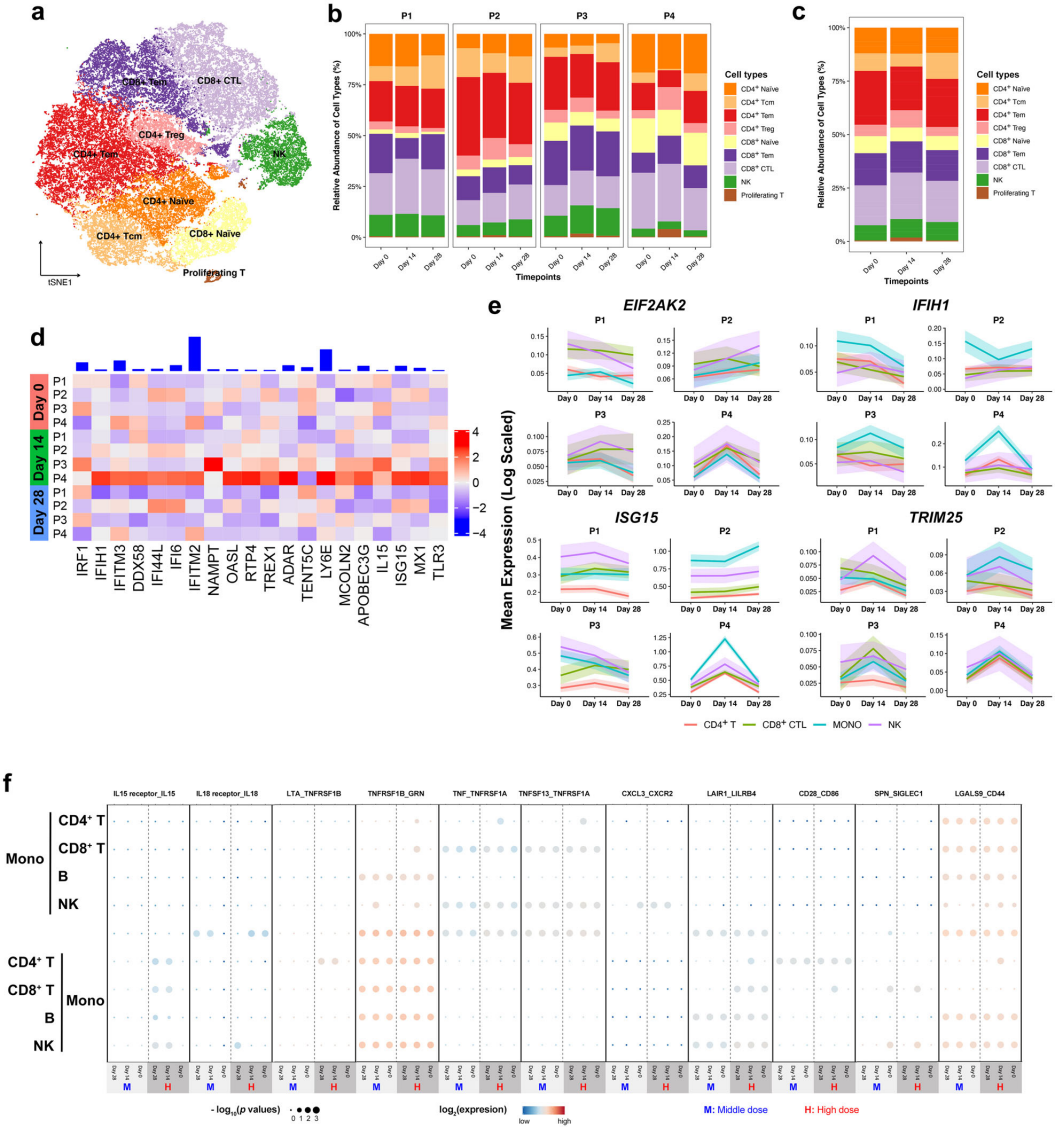
Cellular immune response peaked at day 14 post-vaccination. **a***t*-SNE analysis of NK and T cell subtypes from all participants and timepoints. **b** Proportions of the NK and T cell subtypes in the total NK and T cells from participants at different timepoints by scRNA-seq. **c** Proportions of NK and T cell subtypes of each sample in the total NK and T cells. **d** Heatmap of z-scored mean expression of IFN-response signature (defined as the normalized mean expression of genes in the activation signature in Supplementary Table S3) across T cells from each participant and timepoint. Top, bar plot of total expression of each gene, across all patients. **e** Mean expression of four common ISGs (*EIF2AK2*, *IFIH1*, *ISG15*, and *TRIM25*) in CD4^+^ T, CD8^+^ T, monocytes and NK cells, which are indicated by timepoints and individuals. Shaded area denotes 95% CI of the mean value. **f** Dot plot of the interactions (predicted by ligand/receptor interaction database cellPhoneDB) between monocytes and other immune cell types in the middle- and high-dose group. *P* values are indicated by the circle sizes, as shown in the scale on the bottom. The means of the average expression level of interacting molecule 1 in cluster 1 and interacting molecule 2 in cluster 2 are indicated by the color.

T and NK cells were divided into subsets to further explore the cellular immune response (Fig. 4a). Among the subsets of T cells, the frequency of CD4^+^ Tcms, important cytokine-producing cells, was increased at day 28 post-vaccination (Fig. 4b, c; Supplementary Fig. S4a). It has been reported that CD4^+^ Tcms drive effector responses via interferon (IFN)-γ production and T cell survival and proliferation via IL-2 production^27^, which may account for the elevation of IL-2-secreting CD4^+^ T cells (Supplementary Fig. S4b). In addition, Tregs first rose at day 14 and then decreased at day 28 (Fig. 4b, c), indicating an early reaction towards the immunization that returned to normal levels with time.

Ad5-nCoV was reported to boost rapid cellular immune responses peaking at day 14 after vaccination^16^. IFN-γ has been shown to play a role in the clearance of various viral infections^28^. The frequency of IFN-γ-producing T cells has been widely used as a parameter to assess vaccine-induced responses^29,30^. Thus, we analyzed the T cell reaction at the transcriptomic level through scRNA-seq. We observed inter-patient and inter-timepoint variation in a panel of pro-inflammation and IFN-γ hallmark gene signatures (Fig. 4d). Our analyses showed activation of pro-inflammatory and IFN-γ response-related genes at day 14 after vaccination, which was most pronounced in the high-dose group (especially in P4).

To confirm the trends in protein expression level, IFN-γ enzyme-linked immunospot (ELISpot) assays and intracellular cytokine staining assays (IFN-γ, IL-2, and TNFα) were used to evaluate the specific T cell response. The ELISpot responses peaked at day 14 post-vaccination, beginning from undetectable baseline (Supplementary Fig. S4b). In addition, IFN-γ was detected in CD4^+^ and CD8^+^ T cells after vaccination at day 14 and 28 in both dose groups, and the expression level tended to be higher in the high-dose group than in the middle-dose group. The proportion of cells secreting IL-2 and TNFα was higher in CD4^+^ T cells than in CD8^+^ cells (Supplementary Fig. S4c), indicating that the proportions of polyfunctional phenotypes detected from memory CD4^+^ T cells were higher than those from CD8^+^ T cells.

Interferon-stimulated genes (ISGs) are vital to early viral control. Thus, we examined the expression dynamics of some common ISGs across CD4^+^ T cells, CD8^+^ CTLs, NK cells, and monocytes, which are active mediators of the cellular immune response. Despite being generated in distinct cell types, ISGs including *EIF2AK2* (*PKR*), *FIH1* (*MDA5*), *ISG15*, and *TRIM25* of P4 are characterized by pronounced change in different cell types, peaking at day 14 and decreasing at day 28 (Fig. 4e). Most of those ISGs were reported to target a broad spectrum of RNA and DNA viruses by modulating protein function in the viral life cycle^31–33^. We also noted that high-dose group participants (especially P4) had even broader activation of ISGs, such as *APOBEC3G*, *BST2*, *CGAS*, *DDX58*, *DDX60*, *GBP1*, *GBP2*, *IFIT2*, *IFITM3*, *IRF1*, *MOV10*, *OASL*, and *RTP4* (Supplementary Fig. S4d), indicating highly potent cellular immunity with a higher dose of the vaccine.

To analyze the interaction of immune cell subtypes, we took monocytes into consideration as they were highly prevalent in PBMCs and reported to be involved in the regulation of immune responses^34^. Consistent with a previous report that *TNFSF13* may be beneficial for the recovery of COVID-19 patients^22^, we found that TNF superfamily member and its respective receptors were enriched in high-dose group, particularly at day 14 post-vaccination. Chemokines and cytokines such as *CXCL3*, *IL15*, and *IL18* and their respective receptors were also found to be enriched in high-dose group (Fig. 4f). It was notable that we failed to observe an obvious elevation of inflammatory monocyte-released IL-6 and IL-1β^22^, which were reported to be responsible for inducing an inflammatory storm in severe COVID-19 patients.

### Dissection of T cells and the TCR repertoire

**Fig. 5:**
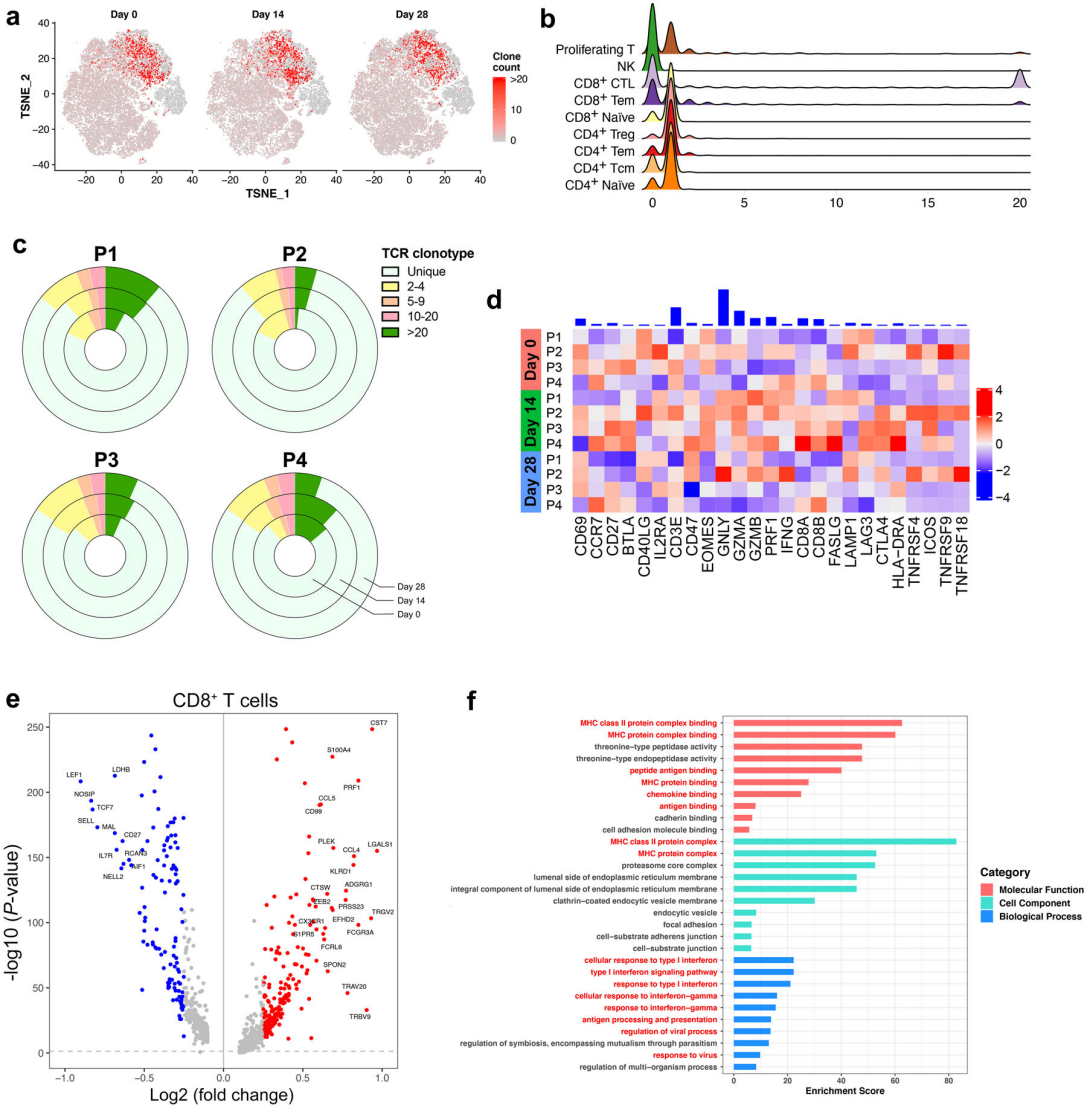
Dissection of T cells and TCR repertoire. **a***t*-SNE showing expanded TCR clones (*n* ≥ 2) in the total T cells indicated by timepoints. **b** Counts of expanded TCR clones in T cell subtypes. **c** The pie plot showing the TCR clone differences across the different timepoints and individuals. **d** Heatmap of z-scored mean expression of T cell activation signature (defined as the normalized mean expression of genes in the activation signature in Supplementary Table S4) across T cells from each participants and timepoints. Top, bar plot of total expression of each gene, across all patients. Expression of T cell activation signature shows variability among individuals. **e** The volcano plot showing the DEGs between CD8^+^ T cells with expanded and unexpanded TCR clones at day 14 post-vaccination. Genes with greatest fold changes and significant *P* values were annotated in the plot. **f** The enriched GO terms of the DEGs between T cells with expanded and unexpanded TCR clones at day 14 post-vaccination. *P* value was derived by a hypergeometric test.

Given concerted IFN responses in T cells, we next evaluated the impact of immunization on T cell distribution. We evaluated the subsets of T and NK cells to observe the temporal change and TCR repertoire of the cell proportions (Fig. 5a).

Here, paired single-cell TCR sequencing (scTCR-seq) were employed to assess TCR clonal expansion at different timepoints. Among all the T cell subtypes, the clonal expansion mainly focused on CD8^+^ CTLs (Fig. 5b). We found that TCRs at day 14 post-vaccination showed a slight shrinkage in unique clonotypes (Fig. 5c). Notably, the clonal changes seemed indistinctive at day 28 after vaccination.

Given that the activation of the T cell response is related to certain upregulated signals, we observed T cell activation genes, including *CD69*, *GZMA*, *GZMB* and *GNLY*, in the whole population of CD8^+^ T cells. Participants at day 14 expressed most T cell activation signals, further confirming the observation of the clinical trial (Fig. 5d). In addition, terminal differentiation (Supplementary Fig. S5a and Table S5) and pro-inflammatory signatures (Supplementary Fig. S5b and Table S6) were also provoked at day 14.

To understand the difference between T cells with unique TCRs and those with expanded TCR clonotypes, we performed DEG analysis on the CD4^+^ and CD8^+^ T cell subgroup from day 14 post-vaccination. As expected, clonal T cells showed more activation- and maturation-related genes, such as *PRF1*, *GZMB*, *DUSP2* and *CST7*, and decreased immature markers, such as *CCR7*, *TCF7*, and *LEF1* (Fig. 5e; Supplementary Fig. S5c). Interestingly, senescence and inhibitory markers, such as *KLRG1* and *EFHD2*, were also increased in clonal T cells (Fig. 5e; Supplementary Fig. S5c), suggesting that the vaccine-elicited antiviral immune response might be controllable. GO analysis revealed even concentrated T cell functional pathways, including antigen processing, antigen presentation, leukocyte adhesion and T cell activation (Fig. 5f). Besides, pathways related to antigen processing and cytotoxicity were enriched in KEGG analysis (Supplementary Fig. S5d). Generally, immunization activates certain T cell subtypes, which function together to mediate a highly controlled cellular immune response.

### Trajectory analysis revealed T cell phenotypes shifting and balanced immune response over time

Naive T cells can differentiate into a number of distinct functional subsets, which enables them to tailor the immune response depending on the type of pathogen and to perform multiple functions during a single infection. Hence, we described the heterogeneity underlying each T cell subtype in a more detailed way by applying trajectory analysis to describe dynamic changes in gene expression.

**Fig. 6:**
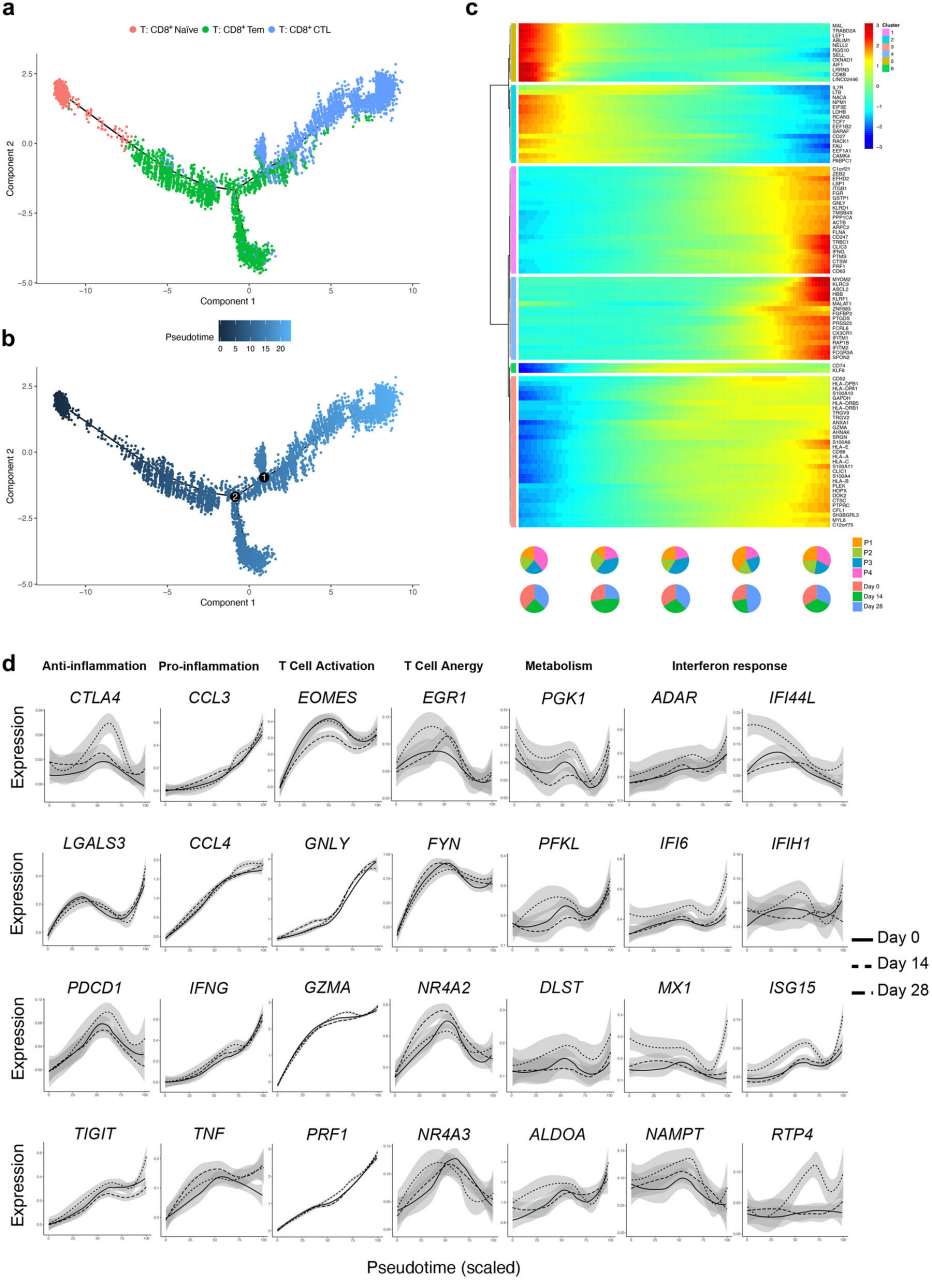
Trajectory analysis revealed the overtime shifting of T cell phenotypes. **a** Pseudotime trajectories for CD8^+^ T cells based on Monocle2, color-coded for the CD8^+^ T cell phenotypes. **b** Pseudotime trajectories for CD8^+^ T cells, color-coded for the pseudotime. **c** Gene expression dynamics along the CD8^+^ T cells lineage. Genes were clustered into 6 gene sets, and each of them was characterized by specific expression profiles. **d** Genes involved in the function and response of T cells modeled along the CD8^+^ T cell lineages at different timepoints. See also “Materials and methods” for constructing single-cell trajectories.

We modeled gene expression pattern along the CD8^+^ T cell lineage. Briefly, naive CD8^+^ T cells connected directly with CD8^+^ Tcms followed by CD8^+^ CTLs (Fig. 6a, b). Unsupervised analysis divided genes into 6 sets. Gene sets 1 and 2 were enriched with genes expressed early in the trajectory, while the others were enriched in the middle or late of the trajectory. From the trajectory along the pseudotime, resting and immature cell markers, including *LEF1*, *SELL* (set 1), *IL7R*, and *TCF7*, were decreased (Fig. 6c). Functional genes involved in cytotoxic function (*PRF1* and *GZMA*), antigen presentation (*CD247* and *HLA*−*DRB5*) and activation-linked co-stimulation (*CD63*) were provoked late along the pseudotime. Some regulatory genes, such as FGR were also increased at the end of the trajectory (Fig. 6c).

Profiling of marker genes, including *EOMES*, *GNLY*, *GZMA*, and *PRF1*, along these trajectories confirmed CD8^+^ T cell functional annotation. Although sharing similar routes, post-vaccination, especially at day 14, showed higher expression of anti-inflammatory (*TIGIT*, *LGALS3*, *PDCD1*, and *CTLA4*) and pro-inflammatory (*TNF*, *IFNG*, *CCL3*, and *CCL4*) markers than pre-vaccination (Fig. 6d). We also found that together with elevated T cell activation response, T cell anergy signatures were also increased, suggesting that CD8^+^ T cells exhibited a balanced phenotype to maintain functional homeostasis (Fig. 6d). Specifically, to investigate the expression of inhibitory markers in the NK and T cells, we analyzed the log2 fold change of gene panel between day 14 and day 28 and observed an overall decrease of inhibitory markers expression from day 14 to day 28 (Supplementary Fig. S6a), suggesting that the emergence of inhibitory markers might be a reaction to maintain a balanced immune response. IFN response marker genes were also increased at day 14 after immunization, indicating activated immune responses. Notably, genes related to metabolism were provoked after injection, such as *PKG1*, *PFKL*, and *ALDOA* involved in glycolysis and *DLST* involved in TCA cycle (Fig. 6d). It might suggest vibrate glycolysis and oxidative phosphorylation in response to the new antigen. We found similar phenotypic shift in the CD4^+^ T cell trajectory, although the changes over time were not as significant as those in CD8^+^ T cells (Supplementary Fig. S6b–e). Overall, gene expression profiling along the trajectories confirmed that T cells of participants exhibited activated but balanced effector functions in a time-dependent manner.

## Discussion

Safe and effective vaccines for COVID-19 are of urgent need to control the pandemic. Current techniques, such as ELISAs and ELISpots, measure the overall responses during immunization, but cannot measure response between certain types of immune cells. Other methods at single cell resolution such as flow cytometry and cytometry by time of flight (cyTOF) require a set of markers to define the state of cells and might introduce artificial bias because of negligible batch effects. Here, we present a comprehensive single-cell analysis of immune dynamics during Ad5-nCoV immunization with minimal sample preparation. We observed temporally aligned alterations in cell type composition, gene expression and immunoglobulin diversity, which collectively provide insights into Ad5-nCoV-elicited immune responses obscured in serological tests. Moreover, we identified potential binding antibodies against SARS-CoV-2 based on scV(D)J-seq data. The most potent one, P3V6-1, exhibited relatively strong S-ECD-binding affinity. Together, our findings suggested that Ad5-nCoV was tolerable and could induce both humoral and cellular immune responses against SARS-CoV-2.

Adenoviral vector-based vaccines are easy to design and produce on a large scale, which provides a promising platform for clinical use. Many human clinical trials have been conducted for adenoviral vector-based vaccines against different infectious agents, including Ebola virus, Zika virus, HIV, and malaria^35–37^. The early results of Ad5-nCoV and other studies demonstrated that adenoviral vector-based vaccines were capable of eliciting pathogen-specific humoral and cellular immunity^38,39^. For humoral immunity, we found that there was an increase in BCR clonal expansion and changes in the utilization of the segments IGHV1-69D, IGKV1D-39, and IGLV5-45. Some of those biased uses of V(D)J genes have been reported to be related to virus-specific antibodies. For example, IGHV1-69D is involved in the human B cell response to dengue virus^40^, and IGHV3-15 was the immunoglobulin gene segment identified in response to an mRNA vaccine^41^. Although IGHV3-53 is the most frequently identified IGHV gene for targeting the S-RBD, we failed to observe a significant change in our participants^23^. A potential reason may be that the preferential function of IGHV3-53 was overwhelmed by other multiple Ad5-nCoV-elicited antibodies targeting non-S-RBD regions.

Antibodies, which can block infection through binding epitopes of antigens, are generated through rearrangement of germline genes, with subsequent somatic mutations that result in a potentially diverse repertoire of antibodies that can combat pathogens. Obtaining full-length antibody IGHVs and IGLVs from individual B cells at scale remains challenging. Though we failed to isolate plasmablasts and B cell subsets by flow cytometry as previous practices did, with the help of optimized antibody selection strategies, we identified five mAbs with special binding affinity with the S-ECD. The most potent mAb, P3V6-1, showed potential neutralizing ability and may serve as a promising intervention for SARS-CoV-2. Our work showed that scRNA-V(D)J-seq has the potential to improve antigen screening and selection. Undoubtedly, both the RBD and ECD of S protein are quite immunogenic, let alone that a study has shown that antibody responses elicited by natural SARS-CoV-2 infection were diverse in epitope recognition of S proteins, including both RBD- and non-RBD-binding neutralizing mAbs^42^. Noticeably, non-RBD-binding neutralizing mAbs were also observed for MERS-CoV^21,43^. It is unclear how non-RBD-directed mAbs block SARS-CoV-2 infection, but there exists a hypothesis that mAbs targeting non-overlapping epitopes, such as the NTD, may prevent escape mutations of the virus and serve as promising “cocktail” therapeutics^21^. Additionally, deeper sequencing of the single-cell libraries may increase the “hit-rate” of predicted antibodies and accuracy of heavy and light chain pairing, since it will enable even more confident identification of B cell subtypes.

For cellular immunity, there is a direct association between the vaccine-elicited T cell response intensity and the capability of virus elimination. Studies have provided evidence that effective prophylactic vaccines against replicating viruses should engage strong cellular T cell-based immunity^8,44^. Although Ad5-nCoV was reported to be capable of stimulating rapid T cell responses, the critical factors of T cell-mediated immune protection against SARS-CoV-2 have not been clearly defined. The primary discovery of cell type composition showed an increase in CD4^+^ Tcms, which are distinguished by a superior proliferation capacity and production of cytokines such as IL-2 and TNFα^45^. CD4^+^ Tcms are required for long-lived immunity and are induced by vaccination strategies, including those against influenza^46^. Consistent with our observation, the proportion of cells secreting IL-2 and TNFα was higher among CD4^+^ T cells than among CD8^+^ cells.

It was reported that adenoviruses activate several innate immune signaling pathways that result in the secretion of a number of pro-inflammatory cytokines^47,48^. Interestingly, these pro-inflammatory cytokines could inform effective immune cell stimulation and result in the induction of robust adaptive cellular immune responses. The results of these studies are consistent with our findings that immunization is associated with increased expression of pro-inflammatory cytokines, such as *TNF*, *CCL3*, *CCL4*, and *IL2*. When performing the trajectory analysis of T cell subtypes, we found both activated signals of pro-inflammation and anti-inflammation, as well as activation and anergy signals of T cells. This result shows that T cells in vaccine recipients, unlike patients with critical COVID-19, are controlled and well ordered. Collectively, these findings help illustrate the possible molecular basis of post-vaccination response, leading to a better understanding of the mechanisms of the T and B cell immune responses.

However, the limited sample size makes it insufficient to confirm the dose-dependent effect of immune responses and the dose of the vaccine in our study. Shrinkage in unique TCR clonotypes is not as significant in these samples, which might be modified if more T cells or TCRs were analysed for each case. Meanwhile, current depth of scRNA-seq we used in this study was not able to distinguish B cell subtypes with limited cell numbers. Therefore, with the develpment of high resolution scRNA-seq and high-quality antigen-specific B-cell sorting, a more comprehensive immune landscape could be depicted and we may evaluate the effectiveness of vaccinaion and detect potential antibodies more efficiently.

Taken together, our results provide single-cell landmarks of major immune cells that help elucidate the tolerability and immunogenicity of Ad5-nCoV. Characterization of phenotype shifting by scRNA-seq can improve our understanding of how the immune repertoire responds to novel microbial pathogens or adenovirus-vectored vaccines, which facilitate the development of vaccine design strategy against pathogens such as SASR-CoV-2.

## Materials and methods

### Study participants

Four participants in this study were enrolled in the phase 1 trial of an Ad5-vectored COVID-19 vaccine funded by the National Key R&D Program of China and CanSino Biologics (NCT04313127; ChiCTR2000030906). This phase 1 trial recruited healthy adults who were free of SARS-CoV-2 infection. The Ad5-vectored COVID-19 vaccine was administered intramuscularly in the arm of the participants. Two participants (P1 and P2) in the middle-dose group received 1 × 10^11^ viral particles, and another two (P3 and P4) in the high-dose group received 1.5 × 10^11^ viral particles in total. Health condition and adverse events during the trial were self-reported and closely monitored by the investigator. The participants, aged between 41 and 46, consisted of two males and two females, and their demographic characteristics are provided in Supplementary Tables S1 and S2. Written informed consent was obtained from each participant, and this study was approved by the Ethics Committee of Jiangsu Provincial Center of Disease Control and Prevention.

### Blood samples for serology tests and PBMC collection

All human blood samples were collected before vaccination and at day 14 and 28 post-vaccination for laboratory assessment and PBMC isolation. Binding antibody responses against the RBD and spike glycoprotein were assessed using ELISA kits (Beijing Wantai BioPharm, Beijing, China). Vaccination-induced neutralizing antibody responses were measured by neutralization tests for both SARS-CoV-2 authentic virus and pseudovirus. Specific T cell responses were quantified with an IFN-γ ELISpot assay. CD4^+^ and CD8^+^ T cell responses were assessed by intracellular cytokine staining assays for IFNγ, IL-2, and TNFα. The generated data of serology tests were shared and agreed upon for publication by the vaccine trial team.

To obtain PBMCs, heparinized venous blood of participants was isolated through Ficoll-Hypaque sedimentation according to standard density-gradient centrifugation methods (GE Healthcare). PBMCs were stored frozen in RPMI 1640 medium supplemented with 20% fetal bovine serum (FBS) and thawed before use. For each sample, the cell viability exceeded 75%.

### ScRNA-V(D)J seq

The single-cell suspensions of scRNA-seq samples were converted to barcoded scRNA-seq libraries using a Chromium Single Cell 5′ Library, Gel Bead and Multiplex Kit, and Chip Kit (10× Genomics). A Chromium Single Cell 5′ v2 Reagent Kit (10x Genomics, 120237) was used to prepare single-cell RNA libraries according to the manufacturer’s protocol. Full-length TCR/BCR V(D)J segments were enriched from amplified cDNA from 5′ libraries via PCR amplification using a Chromium Single-Cell V(D)J Enrichment Kit according to the manufacturer’s protocol (10x Genomics, PN-1000005 and PN-1000016). The mRNA library average sequencing depth aimed for was 10,000 read pairs per cell and 5000 read pairs per cell for the V(D)J libraries.

### Single-cell data alignment and analysis

We demultiplexed and barcoded the sample by using the Cell Ranger Software Suite (v3.1.0) and a command Cell Ranger count. After obtaining each sample, the gene counts were aggregated. Finally, the gene-barcode matrix of all four participants and timepoints was integrated with Seurat^49^ (https://satijalab.org/) and monocle2^50^ (http://cole-trapnell-lab.github.io/monocle-release/docs/).

The TCR/BCR sequences for each single T/B cell were assembled by the Cell Ranger vdj pipeline (v6.0.1), leading to the identification of the CDR3 sequence and the rearranged TCR/BCR gene. Analysis was performed using Loupe V(D)J Browser version 2.0.1 (https://support.10xgenomics.com, 10x Genomics). As described previously^51^, only T cells with at least one TCR α-chain (TRA)and one TCR β-chain (TRB), were remained. For those with more than one TRA or TRB chains assembled, the highest expressed chain (UMI or reads) was regarded as the dominated chain. Each unique dominated TRA and TRB pair was defined as a clonotype. BCR clonotypes were identified similar to TCR. Each unique dominated IGH-IGL/IGK pair was defined as a clonotype. Using these rules, we generated basic sample statistics—such as the read counts, number of clonotypes, frequency of the share of clonotypes in each sample, repertoire richness and diversity for samples. In the post-processing steps, we projected T/B cells with dominant TCR/BCR clonotypes on a *t*-SNE plot using barcode information^52^.

### Differential analysis

Seurat package FindAllMarkers in Seurat (v3.1.5) was used to perform differential analysis between the control and disease groups of the same cell type, the function parameters we used in Seurat v3 are default (Wilcoxon Rank Sum test). For each cluster, DEGs were generated relative to all of the other cells. The threshold for DEGs is logFC > 0.5 and *P* < 0.05.

### Ligand/receptor interaction analysis

To identify potential cellular communications between different cell types, we applied the CellphoneDB v.2.0 algorithm to the scRNA-seq profiles from the middle- and high-dose group^53^. CellphoneDB evaluated the impact of the ligand/receptor interactions based on ligand expression in one cell type and the corresponding receptor expression in another cell type. We focused on the enriched ligand/receptor interactions in Ad5-nCoV participants and selected the ligand/receptor interactions associated with highly significant as well as pairs that were highly expressed.

### Constructing single-cell trajectories

We constructed single-cell trajectory of each sample by using reversed graph embedding method implemented in R Monocle package (version 2.6.3)^50^. Monocle learns the transcriptional changes of single-cells and constructs a trajectory that mainly reflects the progress of cells moving from the starting state. We detected genes that followed similar kinetic trends along the CD8^+^ T cell trajectory as well as CD4^+^ T cell trajectory from the starting state. Hierarchical clustering was applied to cluster genes into five or six subgroups according to the expression patterns.

For the Fig. 6d and Supplementary Fig. S6d, the *x*-axis (pseudotime, scaled) is the conversion value mapped from the pseudotime value of the cell in the time trajectory analysis result to the interval [0,100]. The conversion function is as follows:

$$\begin{array}{l}{\mathrm{pseudotime}}\left( {{\rm{scaled}}} \right)_{\rm{i}} \\= \displaystyle\frac{{{\mathrm{pseudotime}}_{\rm{i}} - {\rm{Min}}\left( {{\mathrm{pseudotime}}} \right)}}{{{\rm{Max}}\left( {{\mathrm{pseudotime}}} \right) - {\rm{Min}}\left( {{\mathrm{pseudotime}}} \right)}} \times 100\end{array}$$

The *y*-axis Expression is the normalized expression level of the gene in each cell, the normalization method:

$${\mathrm{Expression}}_{{\mathrm{ij}}} = {\mathrm{ln}}\left(\frac{{{\rm{count}}_{{\mathrm{ij}}}}}{{{\mathrm{sum}}\left( {{\rm{count}}_{\rm{j}}} \right)}} \ast 10^4{\mathrm{ + 1}}\right)$$

j: each cell; i: every gene; count is the original UMI number

The fitting curve between Gene Expression and Pseudotime (scaled) is fitted by LOESS method.

### Antibody selection strategies

The antibodies were selected and designed based on the following three strategies. A) Cells from P3 and P4 with complete VH and VL domains were filtered based on the criteria mentioned by Xie. et al.^14^. In brief, sequencing reads were aligned to reference V, D, J, and C genes of B cells. Besides, B cell subtypes, IgG subclasses, and somatic hypermutation rate (SHM) were assessed to increase the efficiency of neutralizing mAb identification. We selected 10 antibody heavy chains and 6 antibody light chains with full-length sequences derived from the high-dose group (P3 and P4). For 4 light chains lacking full-length information, we selected human germlines which were reported to have higher frequency for pairing (generally, IgH chains are sufficient to determine most B cell clonal relationships and able to bind to surrogate light chains^54^). B) After immunization, antigen-activated B cells would go through clonal selection and expansion. Those enriched clonotypes, in response to Ad5-nCoV, will be more likely to yield high-affinity SARS-CoV-2 binding and neutralizing antibodies. Combining the facts that P4 had an increased antibody titer of S protein and newly presented repetitive sequences at day 14 and 28, we tried to clone the full-length antibody sequence by PCR. For antibodies that failed to be cloned in full-length, germline information was used to fulfill the antibody light and heavy chain information. C) M Yuan et al.^55^ reported that the antibodies against coronavirus have a certain germline sequence preference. Based on this observation, we chose 6 antibody sequences from P4 for full-length cloning. The sequences of the identified antibodies are provided in Supplementary Dataset S1.

### Recombinant RBD and S-ECD proteins of SARS-CoV-2

S/RBD recombinant proteins were purchased from Huaan Inc. with > 95% purity. Full-length S/RBD protein was expressed in a baculovirus-insect cell system using the DNA sequence encoding the SARS-CoV-2 S protein (S1+S2 ECD) or RBD protein. The protein was expressed with a poly-histidine tag at the C terminus and purified in sterile 20 mM Tris, 300 mM NaCl, 10% glycerol, pH 8.0.

### Antibody expression and purification

Heavy chain- and light chain-encoding plasmids were transiently transfected into HEK293FE cells at a ratio of 1:2. Seven days later, antibodies in culture supernatants were enriched by a Protein A purification column, quantified by a NanoDrop and further validated by SDS-PAGE.

### Antigen-down ELISA

First, 1 µg/mL antigen (RBD-mFc) was coated on ELISA plates at 4 °C overnight. Then, the ELISA plates were blocked with 1% BSA at 37 °C for 1 h. The first antibody dilutions were set up from 10^4^ ng/mL to 10^−3^ ng/mL with a 1:3 series dilution ratio, and the ELISA plates were incubated at 37 °C for 1 h. Goat anti-human IgG-HRP was used as the second antibody for 30 min at 37 °C. TMB color development agents were freshly prepared, used and stopped with 5% H_2_SO_4_. Antibody-binding affinity was assessed at 450 nm absorbance (OD450).

### ELISA

ELISA was performed according to published protocol^21^. Polystyrene microplates (Corning) were coated overnight with 2 μg/mL SARS-CoV-2 RBD protein (Sino Biological). After washed with PBS containing 0.2% Tween 20 (Solarbio Life Sciences), the plates were blocked using 2% BSA (Sigma Aldrich) in PBST for 1 h at 37 °C. Following washing with PBST, serial dilutions of testing antibodies were added to each well and incubated at 37 °C for 1 h. After washing with PBST, horseradish peroxidase (HRP)-conjugated anti-human IgG antibody (Abcam) was added at the dilution of 1:10,000 and incubated at 37 °C for 1 h. After washing, TMB singlecomponent substrate solution (Solarbio Life Sciences) was added to the microplate and incubated at room temperature for 6 min, followed by adding 2 M H_2_SO_4_ to stop the reaction. The absorbance was detected at 450 nm/630 nm. The data was analyzed using GraphPad Prism 8.0.

### Pseudotyped virus neutralization assay

African green monkey kidney Vero E6 cells were cultured in Dulbecco’s modified Eagle’s medium (DMEM) supplemented with 10 % FBS (Invitrogen). SARS-CoV-2 pseudovirus was a gift from Shanghai Key Laboratory of Medical Biodefense, Second Military Medical University. As described before^56^, Vero cells were seeded in 96-well plates and pre-treated with gradient diluted purified antibodies for 1 h. Cells were then infected with pseudovirus at 37 °C. After adsorption for 1 h, the virus was washed away and the cells were cultured in fresh medium. After 24 h, the cells were stained with anti-SARS-CoV-2 mAb (green) to visualize viral protein expression. Infected cells were identified as GFP-positive cells obtained by using fluorescence microscopy (Olympus IX71).

### Statistical analysis

Statistical analysis was performed using GraphPad Prism v8.0. The utilized statistical test is listed in each figure legend. Statistical significance was evaluated using a one-way analysis of variance (ANOVA), **P < 0.01; *P < 0.05; ns, not significance (*P* > 0.05).

## Supplementary information

Supplementary Information

## Data Availability

The accession number for the sequencing raw data in GSA (Genome Sequence Archive in BIG Data Center, Beijing Institute of Genomics, Chinese Academy of Sciences) is HRA000359 (Bioproject Accession number: PRJCA003578); and statements of data and details of information are available at https://github.com/Andrea2280/Immune-Dynamics-During-Ad5-nCoV-Immunization-.
