# Supplementary material for: Integrated single-cell analysis revealed immune dynamics during Ad5-nCoV immunization
Source: Cell Discov. 2021 Aug 10;7:64. doi: 10.1038/s41421-021-00300-2 (PMC8352953; doi:10.1038/s41421-021-00300-2)
Supplement: Supplementary file 1 — Supplementary Information [file 41421_2021_300_MOESM1_ESM.pdf]

Supplementary Information for

Integrated Single-Cell Analysis Revealed Immune Dynamics During Ad5-nCoV Immunization

Author Information:

Qiqi Cao[1#]; Shipo Wu[2#]; Chuanle Xiao[3#]; Shuzhen Chen[1,4,5#]; Xiangyang Chi[2]; Xiuliang Cui[1,4,5]; Hao Tang[6,7]; Wenru Su[3]; Yingfeng Zheng[3]; Jiayong Zhong[3]; Zhaomin Li[8]; Fang Li[8]; Haijia Chen[9]; Lihua Hou[2*]; Hongyang Wang[1,4,5*]; Wen Wen[1*]

[1] Eastern Hepatobiliary Surgery Hospital, Second Military Medical University, Shanghai, China

[2] Beijing Institute of Biotechnology, Beijing, China

[3] State Key Laboratory of Ophthalmology, Zhongshan Ophthalmic Center, Sun Yat-sen University, Guangzhou, China

[4] National Center for Liver Cancer, Second Military Medical University, Shanghai, China

[5] Ministry of Education (MOE) Key Laboratory on Signaling Regulation and Targeting Therapy of Liver Cancer, Second Military Medical University, Shanghai, China

[6] Department of Respiratory and Critical Care Medicine, Changzheng Hospital, Second Military Medical University, Shanghai, China

[7] Department of Critical Care, Wuhan Huoshenshan Hospital, Wuhan, China

[8] HuaAn McAb Biotech Company, Hangzhou, China

[9] Guangzhou SALIAI Stemcell Science and Technology Co., Ltd., Guangzhou, China

# These authors contributed equally to this work.

Key Words: Single cell RNA and V(D)J sequencing, coronavirus disease 2019 (COVID-19), Ad5-based recombinant vaccine (Ad5-nCoV), antibodies

***Correspondence:**

Dr. Wen Wen (wenwen_smmu@163.com), Eastern Hepatobiliary Surgery Hospital, 225 Changhai Road, Shanghai, China. Tel: 86-21-81875363. Fax number: 86-21-65566851.

Dr. Hongyang Wang (hywangk@vip.sina.com), Eastern Hepatobiliary Surgery Hospital, 225 Changhai Road, Shanghai, China. Tel: 86-21-81875361. Fax number: 86-21-65566851.

Prof. Lihua Hou (houlihua@sina.com), Beijing Institute of Biotechnology, Beijing, China

Table of Contents

Supplementary Figures 4

Supplementary Figure S1 4

Supplementary Figure S2 5

Supplementary Figure S3 7

Supplementary Figure S4 9

Supplementary Figure S5. 11

Supplementary Figure S6. 12

Supplementary Table 14

Supplementary Table S1: Demographical information and blood results of vaccine participants 14

Supplementary Table S2: 10X genomics sequencing parameters of each samples 16

Supplementary Table S3: Expression of Type I Interferon response signature (defined as the normalized mean expression of genes 17

Supplementary Table S4: Expression of T cell activation signature (defined as the normalized mean expression of genes 18

Supplementary Table S5: Expression of Type II Interferon response signature (defined as the normalized mean expression of genes 19

Supplementary Table S6: Expression of T cell terminal differentiation signature (defined as the normalized mean expression of genes 20

Supplementary Dataset 21

Supplementary Dataset S1: Gene and antibody sequences of 21 mAb 21

Supplementary Dataset S2: Sequences of Spike-RBD(aa319-591)-mFC 33

Supplementary Figures

Supplementary Figure S1

a

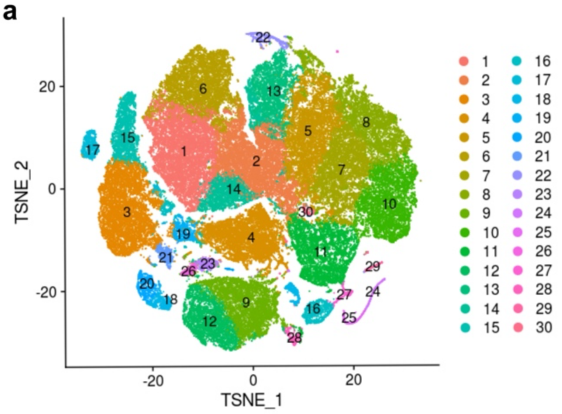

b

Cell types	Sum (cells)	Percentage
$CD4^+$ Naïve	6430	6.95%
$CD4^+$ Tcm	4805	5.20%
$CD4^+$ Tem	12242	13.24%
$CD4^+$ Treg	3113	3.37%
$CD8^+$ Naive	3773	4.08%
$CD8^+$ Tem	7885	8.53%
$CD8^+$ CTL	10652	11.52%
Proliferating T	487	0.53%
NK	4362	4.72%
Naive B	5524	5.97%
Memory B	3613	3.91%
Plasma B	88	0.10%
Aging B	434	0.47%
$CD14^+$ Mono	10011	10.83%
$CD16^+$ Mono	1197	1.29%
Transitional Mono	356	0.39%
cDC1	44	0.05%
cDC2	641	0.69%
pDC	272	0.29%
MEG	2059	2.23%
RBC	780	0.84%
Undefined	13688	14.80%

c

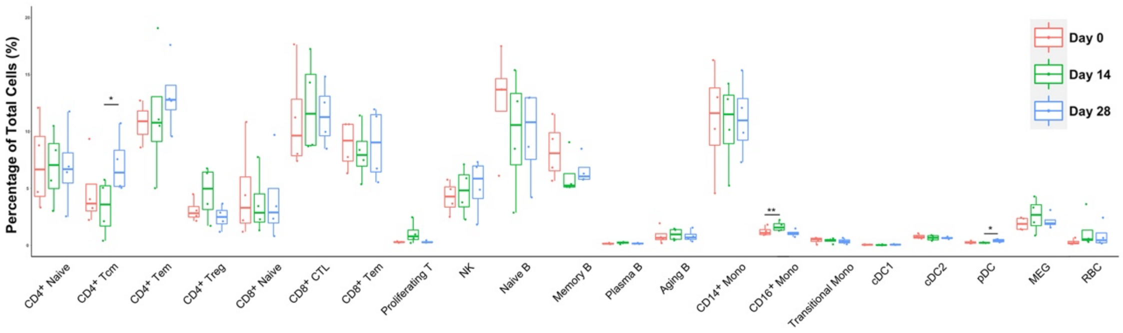

a t-SNE analysis of PBMCs from all participants and timepoints. Cells are primarily unsupervised grouped into 30 clusters.

b Cell counts and percentages of each cell types.

c Proportions of all the cell types in PBMCs from participants in different timepoints by scRNA-seq. Statistical analysis used paired samples t-test. Values are mean ± SD. $^*P < 0.05$, $^{**}P < 0.01$.

Supplementary Figure S2

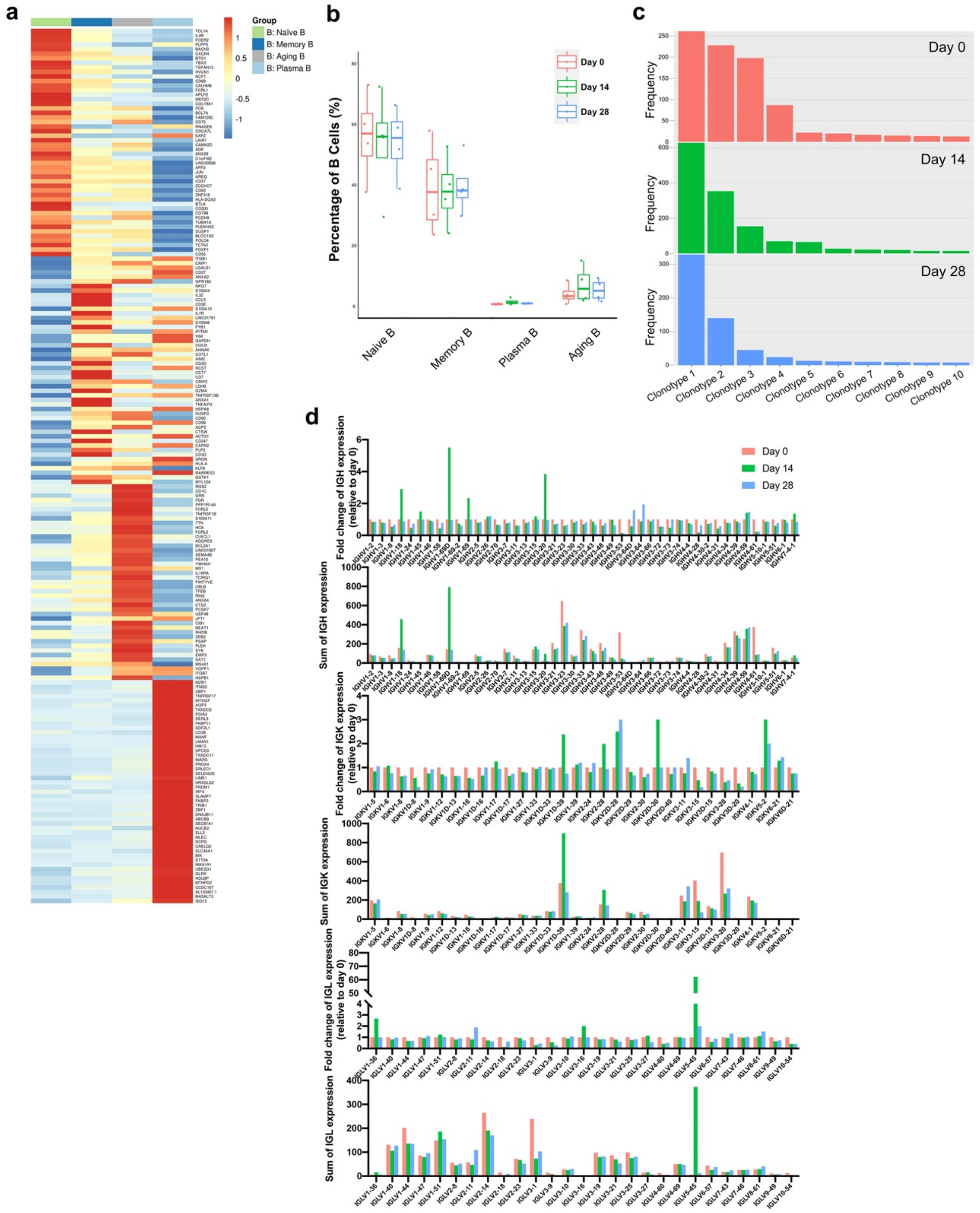

a The heatmaps showing differentially expressed genes (DEGs) upregulated in B cell subtypes.

b Proportions of the B cell subtypes in the total B cells. Statistical analysis used paired t test. Values are mean ± SD. *$P < 0.05$, **$P < 0.01$.

c The frequency of top 10 clonotypes in each timepoints.

d The isotype usage (sum and fold change relative to day 0) of IGH, IGK and IGL at different timepoints.

Supplementary Figure S3

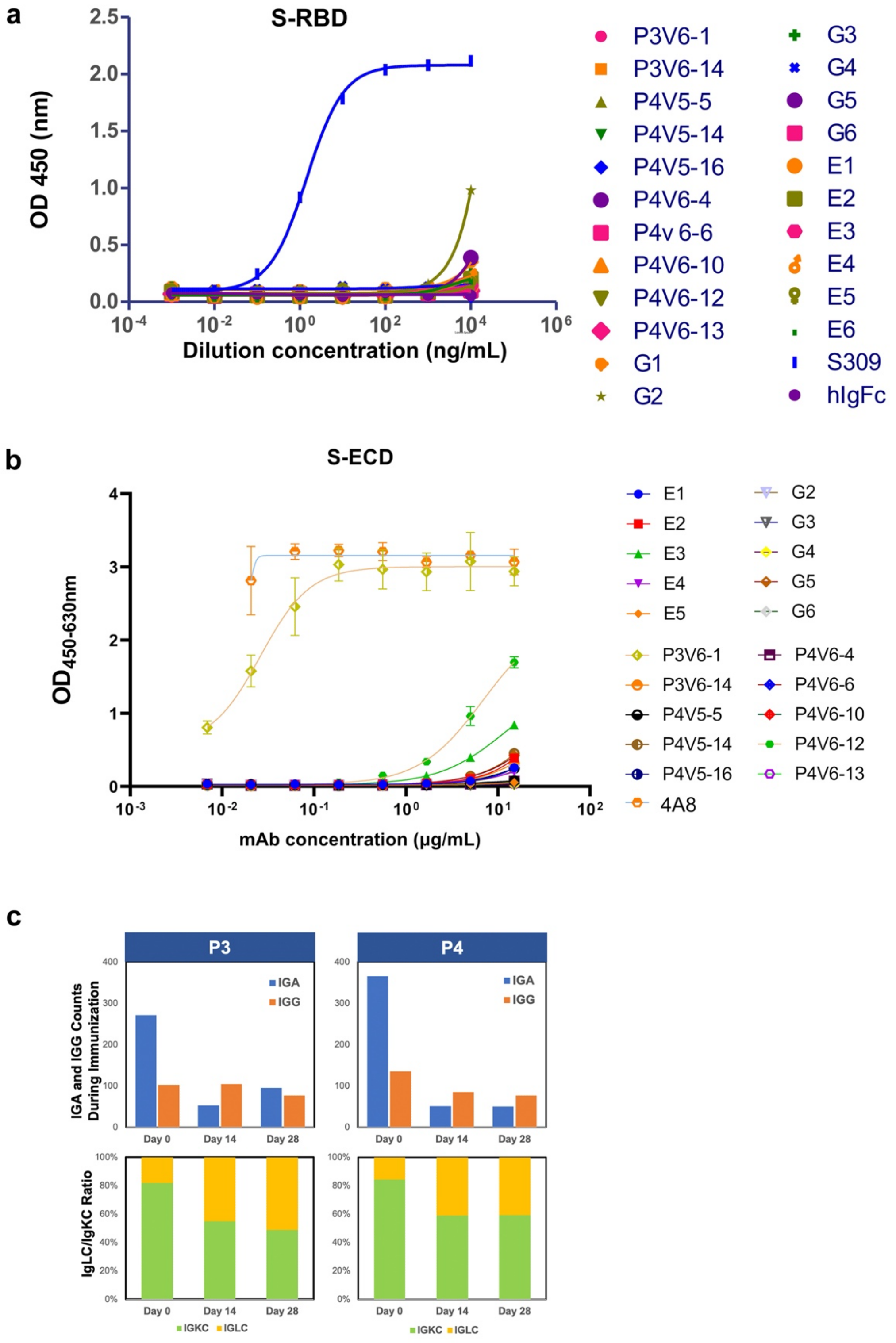

a Binding curves of all synthesized mAbs to S-RBD. S309 is positive control while hIgFC is the negative control.

b Binding curves of all synthesized mAbs to S-ECD. 4A8 is a positive control that was reported to bind the S-ECD of SARS-CoV-2.

c IGA and IGG counts (top) and IgLC/IgKC ratio (bottom) of different timepoints from P3 and P4.

Supplementary Figure S4

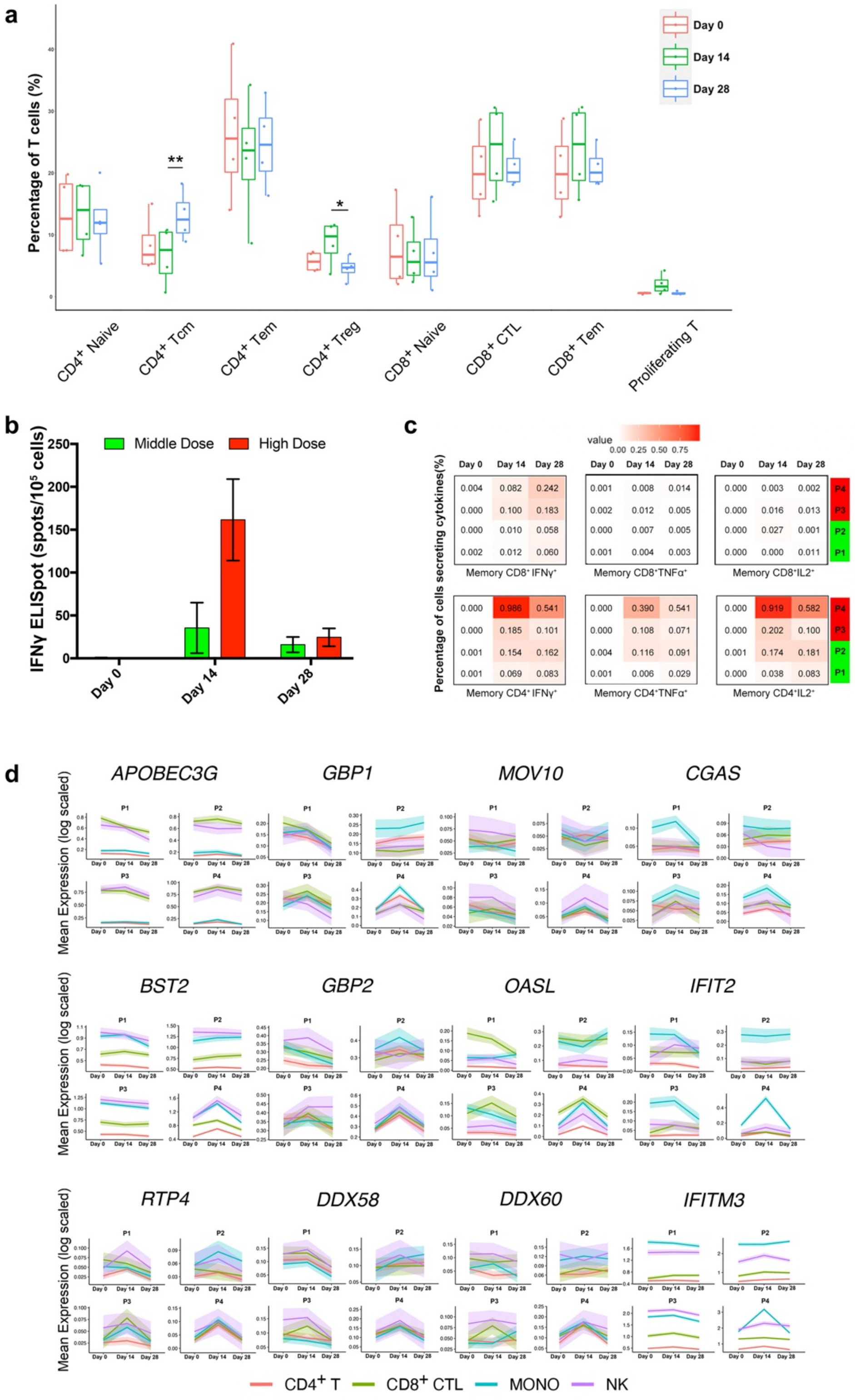

a Proportions of the T and NK cell subtypes in the total T and NK cells. Statistical analysis used paired t test. Values are mean ± SD. $*P < 0.05$, $**P < 0.01$.

b Specific T-cell response measured by ELISpot. The number of specific T cells with secretion of IFNγ (at days 0, 14 and 28) in each participant, and stratified by pre-existing Ad5 neutralizing antibody titres.

c Percentage of cells secreting IFNγ, TNFα, and IL-2 from $CD4^+$ T and $CD8^+$ T cells.

d Mean expression of typical ISGs in $CD4^+$ T, $CD8^+$ T, monocytes and NK cells, which are separated by timepoints and individuals. Shaded area denotes 95% CI of the mean.

Supplementary Figure S5

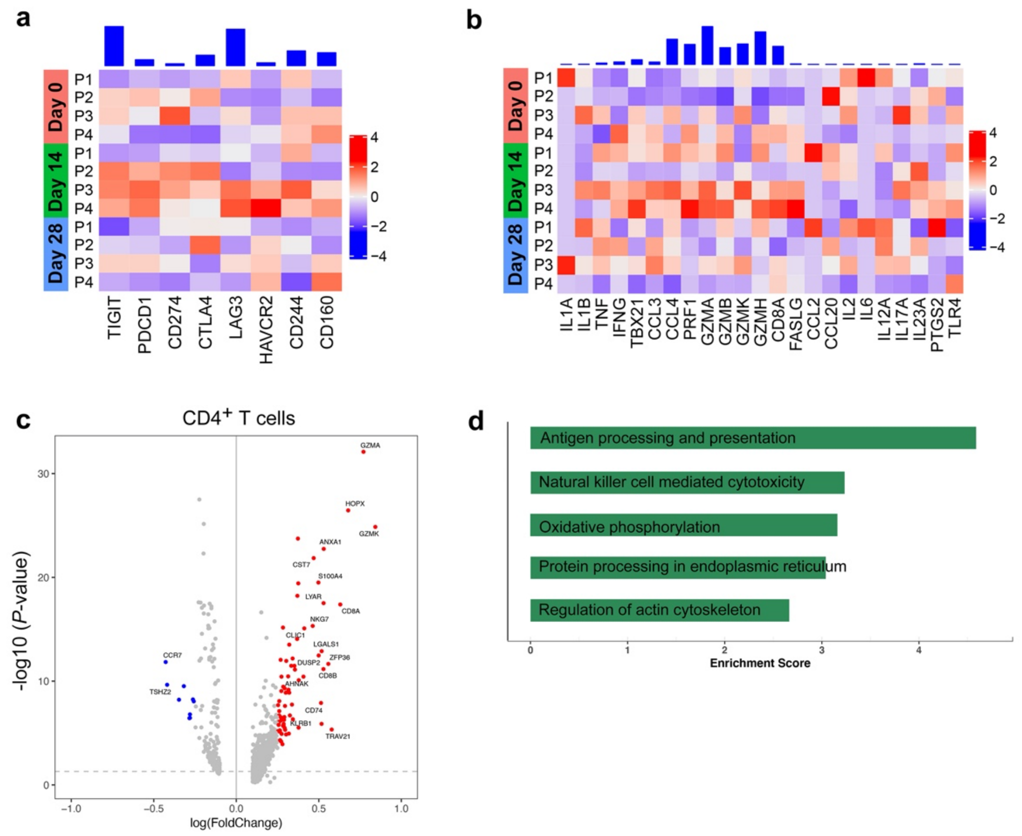

a Heatmap of z-scored mean expression of T cell terminal differentiation signature (defined as the normalized mean expression of genes in the activation signature in Supplementary Table S5) across T cells from each participants and timepoints. Top: Bar plot of total expression of each gene, across all patients. Expression of T cell terminal differentiation signature shows variability across individuals.

b Heatmap of z-scored mean expression of pro-inflammatory signature (defined as the normalized mean expression of genes in the activation signature in Supplementary Table S6) across T cells from each participants and timepoints. Top: Bar plot of total expression of each gene, across all patients. Expression of pro-inflammatory signature shows variability across individuals.

c The volcano plot showing the DEGs between $CD4^{+}$ T cells with expanded and unexpanded TCR clones at day 14 post-vaccination.

d KEGG analysis of the DEGs between T cells with expanded and unexpanded TCR clones at day 14 post-vaccination.

Supplementary Figure S6

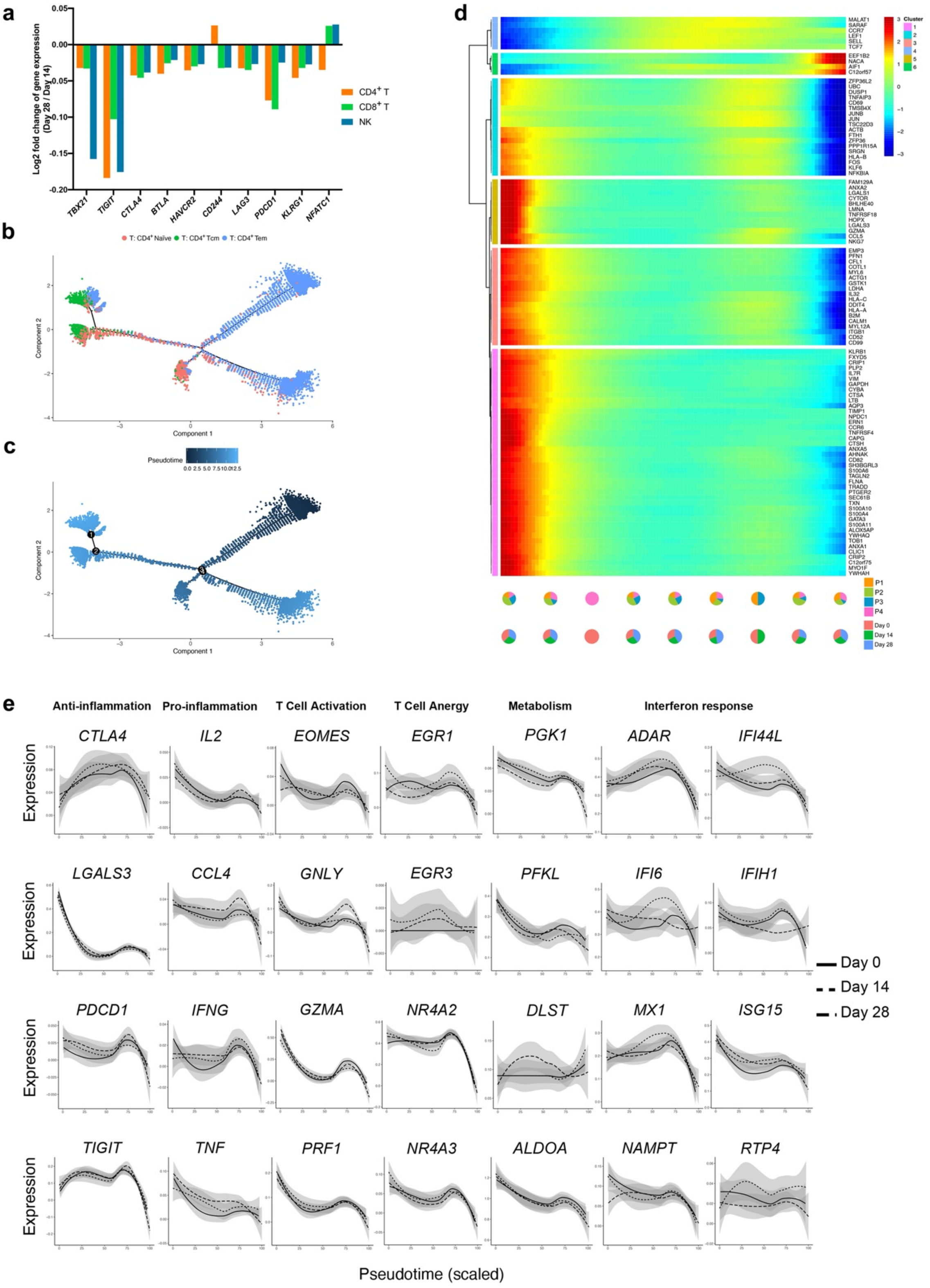

a Pseudotime trajectories for CD4$^+$ T cells based on Monocle2, color-coded for the CD4$^+$ T cell phenotypes.

b Pseudotime trajectories for $CD4^{+}$ T cells, color-coded for the pseudotime.

c Gene expression dynamics along the $CD4^{+}$ T cells lineage. Genes cluster into 6 gene sets, each of them characterized by specific expression profiles.

d Log2 fold change of inhibitory markers between day 14 and day 28 in NK and T cells.

e Genes involved in the function and response of T cells modelled along the $CD4^{+}$ T cell lineages in different timepoints. See also the Method for constructing single-cell trajectories.

Supplementary Table

Supplementary Table S1: Demographical information and blood results of vaccine participants

ID		P1	P2	P3	P4
Dose		Middle	Middle	High	High
Gender		Female	Male	Female	Male
Age		45	46	41	42
SARS-CoV-2 NAb Titer	Day 0	< 8	< 8	< 8	< 8
	Day 14	9	9	33	13
	Day 28	< 8	37	45	72
RBD ELISA Titer	Day 0	< 40	< 40	< 40	64
	Day 14	93	< 40	1112	199
	Day 28	571	138	2579	10694
S ELISA Titer	Day 0	20	20	20	54
	Day 14	104	242	689	1354
	Day 28	246	491	1303	1983
Pseudovirus NAb Titer	Day 0	5	5	5	5
	Day 14	70	5	148	20
	Day 28	16	143	46	284
Ad5 NAb Titer	Day 0	254	232	6	6
	Day 14	1380	8748	100	145
	Day 28	924	8748	107	106
IFNr ELISpot (spots/1e5 cells)	Day 0	1	0	0	0
	Day 14	6	65	114	209
	Day 28	7	25	14	35
Memory $CD8^+IFN\gamma^+$ (%)	Day 0	0.002	0.000	0.000	0.004
	Day 14	0.012	0.010	0.100	0.082
	Day 28	0.060	0.058	0.183	0.242
Memory $CD8^+TNF\alpha^+$ (%)	Day 0	0.001	0.000	0.002	0.001
	Day 14	0.004	0.007	0.012	0.008
	Day 28	0.003	0.005	0.005	0.014
	Day 0	0.000	0.000	0.000	0.000

Memory $CD8^{+}IL2^{+}$ (%)	Day 14	0.000	0.027	0.016	0.003
	Day 28	0.011	0.002	0.013	0.002
Memory $CD4^{+}IFN\gamma^{+}$ (%)	Day 0	0.001	0.001	0.000	0.000
	Day 14	0.069	0.154	0.185	0.986
	Day 28	0.083	0.162	0.101	0.541
Memory $CD4^{+}TNF\alpha^{+}$ (%)	Day 0	0.001	0.004	0.000	0.000
	Day 14	0.006	0.116	0.108	0.390
	Day 28	0.029	0.091	0.071	0.158
Memory $CD4^{+}IL2^{+}$ (%)	Day 0	0.000	0.001	0.000	0.000
	Day 14	0.038	0.174	0.202	0.919
	Day 28	0.083	0.181	0.100	0.582

Abbreviations: IFN, interferon; TNF, tumour necrosis factor; IL, interleukin.

< under detectable baseline

Supplementary Table S2: 10X genomics sequencing parameters of each samples

Patient ID	Timepoint	Estimated Number of Cells	Mean Reads per Cell	Median Genes per Cell
P1	Day 0	6867	68110	1324
	Day 14	7682	55527	1276
	Day 28	8082	54674	1208
P2	Day 0	8384	57276	1202
	Day 14	7229	63621	1285
	Day 28	9603	45795	1190
P3	Day 0	8212	60029	1392
	Day 14	7703	54200	1323
	Day 28	6483	67925	1337
P4	Day 0	7572	54039	1415
	Day 14	6984	60501	1473
	Day 28	7655	54121	1307

Supplementary Table S3: Expression of Interferon response signature (defined as the normalized mean expression of genes)

Patient ID	P1			P2			P3			P4		
**Timepoint**	**Day 0**	**Day 14**	**Day 28**	**Day 0**	**Day 14**	**Day 28**	**Day 0**	**Day 14**	**Day 28**	**Day 0**	**Day 14**	**Day 28**
IRF1	1.00820	0.84289	1.17245	0.76073	0.88340	0.84392	1.23655	1.02655	1.09436	0.83280	1.09289	0.77246
IFIH1	0.13414	0.13349	0.12881	0.13233	0.12137	0.14243	0.12458	0.21887	0.06679	0.13650	0.10771	0.13465
IFITM3	0.84560	0.93071	1.05091	1.43401	0.94434	1.07080	1.18082	1.58309	0.90583	1.13119	0.98612	1.34321
DDX58	0.23472	0.18016	0.19321	0.24178	0.23936	0.21184	0.20048	0.29219	0.13839	0.18943	0.14434	0.18665
IFI44L	0.10579	0.42143	0.13807	0.09634	0.06722	0.34350	0.13722	0.51202	0.07884	0.51051	0.11867	0.13270
IFI6	0.55780	0.83359	0.59020	0.58338	0.52263	0.75417	0.58256	1.07752	0.49880	0.91883	0.50375	0.54318
IFITM2	1.96235	1.99979	1.99874	2.45847	1.99246	2.07260	2.07190	2.59892	1.86129	2.06499	1.95531	2.39346
NAMPT	0.15534	0.15937	0.17591	0.13709	0.16978	0.14610	0.22585	0.16616	0.16374	0.13994	0.16681	0.16055
OASL	0.17101	0.21976	0.11160	0.26112	0.17544	0.21555	0.18350	0.50907	0.08889	0.21737	0.09693	0.17827
RTP4	0.10555	0.05914	0.05991	0.06917	0.10754	0.07783	0.10008	0.20618	0.05651	0.04662	0.04415	0.06532
TREX1	0.02309	0.04798	0.04015	0.05134	0.03355	0.03852	0.04146	0.06204	0.01788	0.03752	0.02201	0.03157
ADAR	0.67533	0.62218	0.65196	0.69334	0.66942	0.63538	0.70141	0.83252	0.66440	0.64330	0.67112	0.65945
TENT5C	0.54075	0.58769	0.60846	0.28390	0.35811	0.51926	0.60498	0.33767	0.66170	0.43421	0.55706	0.34691
LY6E	1.52678	1.63529	1.57032	1.72298	1.52345	1.57072	1.63079	2.18910	1.54468	1.71005	1.57045	1.76020
MCOLN2	0.12809	0.05752	0.12066	0.13745	0.14460	0.08492	0.14575	0.21924	0.12876	0.07504	0.13067	0.13297
APOBEC3G	0.75360	0.54755	0.71980	0.88558	0.68215	0.62639	0.81718	1.13405	0.49378	0.57596	0.63330	0.81825
IL15	0.05152	0.02740	0.05540	0.03101	0.03997	0.03833	0.06600	0.05273	0.03374	0.02454	0.04126	0.02985
ISG15	0.48546	0.64084	0.51472	0.58527	0.48190	0.64248	0.62276	1.03857	0.44385	0.72723	0.53883	0.59224
MX1	0.27429	0.45481	0.26401	0.28800	0.21293	0.40180	0.25116	0.81249	0.21865	0.47562	0.22121	0.32984
TLR3	0.02148	0.01399	0.01522	0.02798	0.01999	0.01901	0.02752	0.04135	0.01011	0.01011	0.01857	0.02551

Supplementary Table S4: Expression of T cell activation signature (defined as the normalized mean expression of genes)

Patient ID	P1			P2			P3			P4		
Timepoint	Day 0	Day 14	Day 28	Day 0	Day 14	Day 28	Day 0	Day 14	Day 28	Day 0	Day 14	Day 28
CD69	1.71648	2.03847	1.94077	1.41654	1.50139	1.93701	1.90391	1.24944	1.88411	1.90219	1.94035	1.47749
CCR7	0.20144	0.40009	0.53638	0.77227	0.24860	0.48596	0.45868	0.82787	0.15154	0.42967	0.41636	0.85267
CD27	0.74480	0.84141	1.02396	0.82803	0.67315	0.92145	1.11484	1.00234	0.55941	0.70273	0.96347	0.95740
BTLA	0.05821	0.06306	0.08292	0.05085	0.06106	0.05920	0.08208	0.08385	0.02469	0.03568	0.05122	0.06434
CD40LG	0.24788	0.24425	0.16967	0.15270	0.13574	0.28594	0.12836	0.17091	0.20503	0.22674	0.10889	0.12598
IL2RA	0.00802	0.01499	0.00974	0.00312	0.01082	0.01119	0.00918	0.00572	0.00996	0.00583	0.01091	0.00396
CD3E	2.63429	2.70226	2.73630	2.72577	2.68704	2.74797	2.70479	2.71095	2.63201	2.70563	2.71496	2.70886
CD47	0.90091	0.94181	0.86841	0.83986	0.92407	0.90536	0.87975	0.98825	0.96417	0.95799	0.76705	0.86345
EOMES	0.54105	0.51162	0.54163	0.47563	0.46403	0.55395	0.55907	0.55567	0.42743	0.43512	0.49845	0.42353
GNLY	3.15915	3.45795	3.15755	3.27290	3.53107	3.51892	3.32752	3.29697	3.40267	3.74371	3.30059	3.10061
GZMA	2.49496	2.54909	2.44710	2.30355	2.60125	2.64510	2.61637	2.55225	2.41290	2.53808	2.42990	2.28270
GZMB	1.71362	1.71165	1.49039	1.82224	2.15004	1.89297	1.71497	2.02915	1.93595	2.07263	1.82312	1.59337
PRF1	1.87849	2.03592	1.72732	2.02710	2.12452	2.17325	1.94236	2.19282	1.77731	2.10111	1.76290	1.79707
IFNG	0.35735	0.60294	0.34807	0.68165	0.66204	0.60731	0.51949	0.53564	0.35628	0.82059	0.51851	0.46813
CD8A	1.68600	1.74903	1.76253	1.84830	1.77796	1.86460	1.90571	2.06191	1.76027	1.65562	1.76894	1.84566
CD8B	1.33602	1.64937	1.73461	1.89964	1.49134	1.75232	1.78427	1.97791	1.35203	1.56104	1.70275	1.98326
FASLG	0.10174	0.06547	0.09226	0.11543	0.14299	0.11975	0.14810	0.19396	0.05409	0.10607	0.09703	0.10647
LAMP1	0.99245	1.06135	0.87974	0.86182	1.01207	0.96763	0.83136	0.86086	0.99644	1.04186	0.89152	0.83298
LAG3	0.68794	0.64003	0.59110	0.51902	0.65576	0.57209	0.70824	0.71928	0.64396	0.60320	0.61873	0.49421
CTLA4	0.02726	0.04922	0.03181	0.01704	0.02289	0.07753	0.08211	0.07806	0.03941	0.06489	0.04083	0.04680
HLA.DRA	0.50783	0.78848	0.66032	0.70657	0.56372	0.81490	1.02737	1.18742	0.60936	0.66835	0.91706	0.76646
TNFRSF4	0.02704	0.14962	0.03096	0.01671	0.01828	0.15130	0.03535	0.02946	0.02262	0.14751	0.01819	0.02825
ICOS	0.05900	0.10550	0.09530	0.07118	0.06892	0.13357	0.12960	0.10808	0.07429	0.10214	0.07050	0.07685
TNFRSF9	0.02596	0.11279	0.01738	0.05127	0.01800	0.08639	0.00512	0.05769	0.01391	0.07270	0.01701	0.01997
TNFRSF18	0.01208	0.33197	0.02355	0.03353	0.00573	0.36090	0.02445	0.02282	0.02340	0.46554	0.01180	0.01987

Supplementary Table S5: Expression of T cell terminal differentiation signature (defined as the normalized mean expression of genes)

Patient ID	P1			P2			P3			P4		
Timepoint	Day 0	Day 14	Day 28	Day 0	Day 14	Day 28	Day 0	Day 14	Day 28	Day 0	Day 14	Day 28
TIGIT	0.28457	0.43261	0.43525	0.37954	0.29340	0.54285	0.53348	0.49085	0.21284	0.36146	0.43312	0.31640
PDCD1	0.05924	0.08287	0.06996	0.04469	0.05406	0.08853	0.10508	0.10106	0.05609	0.06314	0.07883	0.05082
CD274	0.01867	0.02347	0.03254	0.01613	0.02183	0.02954	0.02812	0.02361	0.02331	0.01916	0.02330	0.01692
CTLA4	0.09866	0.15701	0.10458	0.06675	0.10756	0.17826	0.13987	0.12175	0.12467	0.18451	0.07766	0.10296
LAG3	0.41419	0.28302	0.39298	0.34738	0.37041	0.26883	0.50188	0.52782	0.37496	0.29459	0.39208	0.29506
HAVCR2	0.02193	0.01704	0.02149	0.03343	0.02609	0.02872	0.04892	0.07058	0.02381	0.04067	0.04234	0.04641
CD244	0.18075	0.15884	0.18190	0.18007	0.18768	0.14614	0.20745	0.18119	0.14678	0.16954	0.15873	0.13365
CD160	0.10817	0.05635	0.19030	0.23586	0.10616	0.06620	0.16794	0.22731	0.10058	0.07088	0.17947	0.26011

Supplementary Table S6: Expression of pro-inflammatory signature (defined as the normalized mean expression of genes)

Patient ID	P1			P2			P3			P4		
Timepoint	Day 0	Day 14	Day 28	Day 0	Day 14	Day 28	Day 0	Day 14	Day 28	Day 0	Day 14	Day 28
IL1B	0.02264	0.01849	0.04414	0.01061	0.00773	0.00603	0.03899	0.00883	0.04448	0.01485	0.02307	0.01420
TNF	0.11995	0.13610	0.20020	0.08682	0.22177	0.15649	0.24093	0.15446	0.19686	0.22624	0.19902	0.10508
IFNG	0.19177	0.25330	0.20004	0.44752	0.36894	0.27765	0.35926	0.37867	0.19364	0.38907	0.30944	0.27932
TBX21	0.51928	0.42439	0.54715	0.53230	0.52907	0.45901	0.58773	0.66198	0.53052	0.45335	0.53140	0.41496
CCL3	0.19144	0.20548	0.26703	0.34302	0.32598	0.23816	0.41692	0.32895	0.21843	0.36798	0.40535	0.22619
CCL4	1.27653	1.30311	1.70566	1.36431	1.80828	1.35560	1.94258	1.64382	1.62425	1.55272	1.63129	1.16050
PRF1	1.31417	1.13227	1.26173	1.56790	1.48183	1.32217	1.49575	1.76447	1.20424	1.26390	1.28835	1.27614
GZMA	1.88693	1.64152	1.93587	1.84416	1.93245	1.80660	2.16211	2.17753	1.78671	1.69206	1.91532	1.72525
GZMB	1.15690	0.85483	1.03555	1.37049	1.48749	1.05899	1.27840	1.59487	1.32380	1.21233	1.30940	1.08508
GZMK	1.42051	1.31447	1.66820	1.08601	1.06699	1.39835	1.83064	1.39927	1.28262	1.13509	1.63180	1.18604
GZMH	1.70387	1.41996	1.50485	1.88676	1.97238	1.62691	1.78190	2.08093	1.88518	1.71566	1.66951	1.71593
CD8A	1.17431	1.01633	1.31912	1.43807	1.21223	1.14938	1.49336	1.67813	1.21820	0.99396	1.34552	1.35892
FASLG	0.05052	0.02105	0.05169	0.06887	0.06883	0.04645	0.08838	0.12475	0.02553	0.04219	0.05287	0.05524
CCL2	0.00000	0.00000	0.00000	0.00000	0.00057	0.00000	0.00000	0.00000	0.00053	0.00000	0.00000	0.00000
CCL20	0.00292	0.00739	0.00289	0.00165	0.00496	0.00442	0.00183	0.00219	0.00149	0.00507	0.00336	0.00184
IL2	0.01575	0.01168	0.01569	0.00508	0.01161	0.01190	0.00914	0.00369	0.01618	0.00769	0.01467	0.00402
IL6	0.00076	0.00000	0.00000	0.00000	0.00000	0.00000	0.00000	0.00000	0.00060	0.00000	0.00000	0.00000
IL12A	0.01038	0.00693	0.00621	0.00673	0.00895	0.00494	0.00505	0.00569	0.01121	0.01094	0.00910	0.00538
IL17A	0.00088	0.00041	0.00273	0.00036	0.00011	0.00105	0.00222	0.00000	0.00084	0.00084	0.00149	0.00000
IL23A	0.05363	0.07979	0.08547	0.06933	0.06590	0.10864	0.09303	0.08104	0.04561	0.08988	0.07378	0.05159
PTGS2	0.00091	0.00197	0.00000	0.00046	0.00000	0.00000	0.00157	0.00192	0.00400	0.00031	0.00000	0.00000
TLR4	0.00218	0.00052	0.00302	0.00172	0.00352	0.00177	0.00218	0.00340	0.00000	0.00159	0.00050	0.00380

Supplementary Dataset

Supplementary Dataset S1: Gene and antibody sequences of 21 mAb

>P3V6-1 VH
CAGGTGCAGCTGGTGCAGTCTGGGGCTGAGGTGAAGAAGCCTGGGGCCTCAGTG
AAGGTTTCCTGCAAGGCATCTGGATACACCTTCACCAGCTACTATATGCACTGGG
TGCGACAGGCCCCTGGACAAGGGCTTGAGTGGATGGGAATAATCAACCCTAGTG
GTGGTAGCACAAGCTACGCACAGAAGTTCCAGGGCAGAGTCACCATGACCAGGG
ACACGTCCACGAGCACAGTCTACATGGAGCTGAGCAGCCTGAGATCTGAGGACA
CGGCCGTGTATTACTGTGCGAGAGCGCCTTATTACGATTTTTGGAGTGGTTCTTTG
CCGATTGATATCTGGGGCCAAGGGACAATGGTCACCGTCTCTTCA

QVQLVQSGAEVKKPGASVKVSCKASGYTFTSYYMHWVRQAPGQGLEWMGIINPSG
GSTSYAQKFQGRVTMTRDTSTSTVYMELSSLRSEDTAVYYCARAPYYDFWSGSLPID
IWGQGTMVTVSS

>P3V6-1 VL
GAAATAGTGATGACGCAGTCTCCAGCCACCCTGTCTGTGTCTCCAGGGGAAAGA
GCCACCCTCTCCTGCAGGGCCAGTCAGAGTGTTAGCAGCAACTTAGCCTGGTACC
AGCAGAAACCTGGCCAGGCTCCCAGGCTCCTCATCTATGGTGCATCCACCAGGG
CCACTGGTATCCCAGCCAGGTTCAGTGGCAGTGGGTCTGGGACAGAGTTCACTCT
CACCATCAGCAGCCTGCAGTCTGAAGATTTTGCAGTTTATTACTGTCAGCAGTAT
AATAACTGGCCAATCACCTTCGGCCAAGGGACACGACTGGAGATTAAA

EIVMTQSPATLSVSPGERATLSCRASQSVSSNLAWYQQKPGQAPRLLIYGASTRATGI
PARFSGSGSGTEFTLTISSLQSEDFAVYYCQQYNNWPITFGQGTRLEIK

>P3V6-14 VH
GAGGTGCAGCTGTTGGAGTCTGGGGGAGGCTTGGTACAGCCTGGGGGGTCCCTG
AGACTCTCCTGTGCAGCCTCTGGATTCACCTTTAGCAGCTATGCCATGAGCTGGG
TCCGCCAGGCTCCAGGGAAGGGGCTGGAGTGGGTCTCAGCTATTAGTGGTAGTG
GTGGTAGCACATACTACGCAGACTCCGTGAAGGGCCGGTTCACCATCTCCAGAG
ACAATTCCAAGAACACGCTGTATCTGCAAATGAACAGCCTGAGAGCCGAGGACA
CGGCCGTATATTACTGTGCGAAAGCCCCCCCTGTAGTAGTACCAGCTGCGCACG
GGTGGTTCGACCCCTGGGGCCAAGGAACCCTGGTCACCGTCTCCTCA

EVQLLESGGGLVQPGGSLRLSCAASGFTFSSYAMSWVRQAPGKGLEWVSAISGSGG
STYYADSVKGRFTISRDNSKNTLYLQMNSLRAEDTAVYYCAKAPPVVVPAAHGWF
DPWGQGTLVTVSS

>P3V6-14 VL

CAGTCTGTGCTGACGCAGCCGCCCTCAGTGTCTGGGGCCCCAGGGCAGAGGGTC
ACCATCTCCTGCACTGGGAGCAGCTCCAACATCGGGGCAGGTTATGATGTACACT
GGTACCAGCAGCTTCCAGGAACAGCCCCCAAACTCCTCATCTATGGTAACAGCA
ATCGGCCCTCAGGGGTCCCTGACCGATTCTCTGGCTCCAAGTCTGGCACCTCAGC
CTCCCTGGCCATCACTGGGCTCCAGGCTGAGGATGAGGCTGATTATTACTGCCAG
TCCTATGACAGCAGCCTGAGTGGTTGGGTGTTCGGCGGAGGGACCAAGCTGACC
GTCCTAG

QSVLTQPPSVSGAPGQRVTISCTGSSSNIGAGYDVHWYQQLPGTAPKLLIYGNSNRPS
GVPDRFSGSKSGTSASLAITGLQAEDEADYYCQSYDSSLSGWVFGGGTKLTVL

>P4V5-5 VH
GAAGTGCAGCTGGTGGAGTCTGGGGGAGGCTTGGTACAGCCTGGCAGGTCCCTG
AGACTCTCCTGTGCAGCCTCTGGATTCACCTTTGGTGATTATGCCATGCACTGGG
TCCGGCAAGCTCCAGGGAAGGGCCTGGAGTGGGTCTTGGGTATTAGTTGGAATA
GTGGTAGCATAGGCTATGCGGACTCTGTGAAGGGCCGATTCACCATCTCCAGAG
ACAACGCCAAGAACTCCCTGTATCTGCAAATGAACAGTCTGAGAGCTGAGGACA
CGGCCTTGTATTACTGTGCAAAAGATGTTAATTTTGACAATTACTATGATAGTAG
TTTAATGGGGGGCTACTTTGACTACTGGGGCCAAGGAACCCTGGTCACCGTCTCC
TCA

EVQLVESGGGLVQPGRSLRLSCAASGFTFGDYAMHWVRQAPGKGLEWVLGISWNS
GSIGYADSVKGRFTISRDNAKNSLYLQMNSLRAEDTALYYCAKDVNFDNYYDSSLM
GGYFDYWGQGTLVTVSS

>P4V5-5 VL
CAGCTTGTGCTGACTCAATCGCCCTCTGCCTCTGCCTCCCTGGGAGCCTCGGTCA
AGCTCACCTGCACTCTGAGCAGTGGGCACAGCAGCTACGCCATCGCATGGCATC
AGCAGCAGCCAGAGAAGGGCCCTCGGTACTTGATGAAGCTTAACAGTGATGGCA
GCCACAGCAAGGGGGACGGGATCCCTGATCGCTTCTCAGGCTCCAGCTCTGGGG
CTGAGCGCTACCTCACCATCTCCAGCCTCCAGTCTGAGGATGAGGCTGACTATTA
CTGTCAGACCTGGGACACTGGCATTCGAGTGTTCGGCGGAGGGACCAAGCTGAC
CGTCCTAG

QLVLTQSPSASASLGASVKLTCTLSSGHSSYAIAWHQQQPEKGPRYLMKLNSDGSHS
KGDGIPDRFSGSSSGAERYLTISSLQSEDEADYYCQTWDTGIRVFGGGTKLTVL

>P4V5-14 VH
GAGGTGCAGCTGTTGGAGTCTGGGGGAGGCTTGGTACAGCCTGGGGGGTCCCTG
AGACTCTCCTGTGCAGCCTCTGGATTCACCTTTAGCAGCTATGCCATGAGCTGGG
TCCGCCAGGCTCCAGGGAAGGGGCTGGAGTGGGTCTCAGTTATTTATAGCGGTG
ATGATAGCACATACTATGCAGACTCCGTGAAGGGCCGGTTCACCATCTCCAGAG

ATTCCTCCAAGTCCACGCTGTTTCTGCAAATGAACAGCCTGAGAGCCGACGACAC
GGCCGTATATTACTGTGCGAAGGGGGTAGTAGTACCAGCTGAGTGGGGCCAAGG
AACCCTGGTCACCGTCTCCTCA

EVQLLESGGGLVQPGGSLRLSCAASGFTFSSYAMSWVRQAPGKGLEWVSVIYSGDD
STYYADSVKGRFTISRDSSKSTLFLQMNSLRADDTAVYYCAKGVVVPAEWGQGTLV
TVSS

>P4V5-16 VH
GAGGTGCAGCTGGTGGAGTCTGGGGGAGGCTTGGTCCAGCCTGGGGGGTCCCTG
AGACTCTCCTGTGCAGCCTCTGGATTCACCTTTAGTAGCTATTGGATGAGCTGGG
TCCGCCAGGCTCCAGGGAAGGGGCTGGAGTGGGTGGCCAACATAAAGCAAGATG
GAAGTGAGAAATACTATGTGGACTCTGTGAAGGGCCGATTCACCATCTCCAGAG
ACAACGCCAAGAACTCACTGTATCTGCAAATGAACAGCCTGAGAGCCGAGGACA
CGGCTGTGTATTACTGTGCGAGAGAGGTGCCCGCGTACACGCGTCGGGTCTTCA
TGTCGGGGGGGGGGGGTATGGACGTCTGGGGGCAAGGGACCACGGTCACCGTCT
CCTCA

EVQLVESGGGLVQPGGSLRLSCAASGFTFSSYWMSWVRQAPGKGLEWVANIKQDG
SEKYYVDSVKGRFTISRDNAKNSLYLQMNSLRAEDTAVYYCAREVPAYTRRVFMSG
GGGMDVWGQGTTVTVSS

>P4V6-4 VH
CAGGTGCAGCTGCAGGAGTCGGGCCCAGGACTGGTGAAGCCTTCGGAGACCCTG
TCCCTCACCTGCACTGTCTCTGGTGGCTCCATCAGTAGTTACTACTGGAGCTGGA
TCCGGCAGCCCCCAGGGAAGGGACTGGAGTGGATTGGGTATATCTATTACAGTG
GGAGCACCAACTACAACCCCTCCCTCAAGAGTCGAGTCACCATATCAGTAGACA
CGTCCAAGAACCAGTTCTCCCTGAAGCTGAGCTCTGTGACCGCTGCGGACACGGC
CGTGTATTACTGTGCGAGAGCCCGTTACGATATTTTGGGTGAATGGGGAAAAA
ATGATGCTTTTGATATCTGGGGCCAAGGGACAATGGTCACCGTCTCTTCA

QVQLQESGPGLVKPSETLSLTCTVSGGSISSYYWSWIRQPPGKGLEWIGYIYYSGSTN
YNPSLKSRVTISVDTSKNQFSLKLSSVTAADTAVYYCARARYDILGEWGKNDAFDIW
GQGTMVTVSS

>P4V6-4 VL
GACATCCAGATGACCCAGTCTCCTTCCACCCTGTCTGCATCTGTAGGAGACAGAG
TCACCATCACTTGCCGGGCCAGTCAGAGTATTAGTAGCTGGTTGGCCTGGTATCA
GCAGAAACCAGGGAAAGCCCCTAAGCTCCTGATCTATGATGCCTCCAGTTTGGA
AAGTGGGGTCCCATCAAGGTTCAGCGGCAGTGGATCTGGGACAGAATTCACTCT
CACCATCAGCAGCCTGCAGCCTGATGATTTTGCAACTTATTACTGCCAACAGTAT
AATAGTTATTCTCCGATGTACACTTTTGGCCAGGGGACCAAGCTGGAGATCAAA

DIQMTQSPSTLSASVGDRVTITCRASQSISSWLAWYQQKPGKAPKLLIYDASSLESGV
PSRFSGSGSGTEFTLTISSLQPDDFATYYCQQYNSYSPMYTFGQGTKLEIK

>P4V6-6 VH
CAGGTTCAGCTGGTGCAGTCTGGAGCTGACGTGAAGAAGCCTGGGGCCTCAGTG
AAGGTCTCCTGCCGAGCTTCTGGTTACTCTTTTAGCAGCAATGGTCTTAACTGGG
TGCGACAGGCCCCTGGACAAGGGCTTGAGTGGATGGGATGGATCAGCGGTTACA
ATGGTAACACAAACTATGCACAGAAGTTTCAGGGCAGAGTCACCATGACCACAG
ACACATCCACGAGCACAGCCTACATGGAGCTGAGGAGCCTGAGATCTGACGACA
CGGCCATCTATTACTGTGCGAGAGGCGGCCAAGTCTACCCTCCTAGAGAGTTTG
ACTACTGGGGCCAAGGAACCCTGGTCACCGTCTCCTCA

QVQLVQSGADVKKPGASVKVSCRASGYSFSSNGLNWVRQAPGQGLEWMGWISGY
NGNTNYAQKFQGRVTMTTDTSTSTAYMELRSLRSDDTAIYYCARGGQVYPPREFDY
WGQGTLVTVSS

>P4V6-10 VH
CAGGTGCAGCTGGTGCAGTCTGGGGCTGAGGTGAAGAAGCCTGGGGCCTCAGTG
AAGGTTTCCTGCAAGGCATCTGGATACACCTTCACCAGCTACTATATGCACTGGG
TGCGACAGGCCCCTGGACAAGGGCTTGAGTGGATGGGAATAATCAACCCTAGTG
GTGGTAGCACAAGCTACGCACAGAAGTTCCAGGGCAGAGTCACCATGACCAGGG
ACACGTCCACGAGCACAGTCTACATGGAGCTGAGCAGCCTGAGATCTGAGGACA
CGGCCGTGTATTACTGTGCGAGAGGACGTTATGATTACGTTTGGGGGAGTTATCG
TTATACCGGTGATGTTTTTGACTACTGGGGCCAAGGAACCCTGGTCACCGTCTCC
TCA

QVQLVQSGAEVKKPGASVKVSCKASGYTFTSYYMHWVRQAPGQGLEWMGIINPSG
GSTSYAQKFQGRVTMTRDTSTSTVYMELSSLRSEDTAVYYCARGRYDYVWGSYRY
TGDVFDYWGQGTLVTVSS

>P4V6-10 VL
GAAATTGTGTTGACACAGTCTCCAGCCACCCTGTCTTTGTCTCCAGGGGAAAGAG
CCACCCTCTCCTGCAGGGCCAGTCAGAGTGTTAGCAGCTACTTAGCCTGGTACCA
ACAGAAACCTGGCCAGGCTCCCAGGCTCCTCATCTATGATGCATCCAACAGGGC
CACTGGCATCCCAGCCAGGTTCAGTGGCAGTGGGTCTGGGACAGACTTCACTCTC
ACCATCAGCAGCCTAGAGCCTGAAGATTTTGCAGTTTATTACTGTCAGCAGCGTA
GCAAGTGGCCGGACACTTTTGGCCAGGGGACCAAGCTGGAGATCAAA

EIVLTQSPATLSLSPGERATLSCRASQSVSSYLAWYQQKPGQAPRLLIYDASNRATGI
PARFSGSGSGTDFTLTISSLEPEDFAVYYCQQRSKWPDTFGQGTKLEIK

>P4V6-12 VH
CAGGTTCAGCTGGTGCAGTCTGGAGCTGAGGTGAAGAAGCCTGGGGCCTCAGTG
AAGGTCTCCTGCAAGGCTTCTGGTTACACCTTTACCAGCTATGGTATCAGCTGGG
TGCGACAGGCCCCTGGACAAGGGCTTGAGTGGATGGGATGGCTGAGCGTGTTTA
ATGGTAACACAAACTATGCACAGAAGTTTCAGGGCAGAGTCACCCTGACCACAG
ACACATCCACGAGCACAGCCTACATGGAGCTGAGGAGCCTGAGATCTGAGGACA
CGGCCGTGTATTACTGTGCGAAGGTGTCTGTGGCTGGTAGAGCTCCTGACTTTGA
CTATTGGGGCCAAGGAACCCTGGTCACCGTCTCCTCA

QVQLVQSGAEVKKPGASVKVSCKASGYTFTSYGISWVRQAPGQGLEWMGWLSVFN
GNTNYAQKFQGRVTLTTDTSTSTAYMELRSLRSEDTAVYYCAKVSVAGRAPDFDY
WGQGTLVTVSS

>P4V6-13 VH
CAGGTCCAGCTTGTGCAGTCTGGGGCTGAGGTGAAGAAGCCTGGGGCCTCAGTG
AAGGTTTCCTGCAAGGCTTCTGGATACACCTTCACTAGCTATGCTATGCATTGGG
TGCGCCAGGCCCCCGGACAAAGGCCTGAGTGGATGGGATGGATCTCCGTGGACA
ATGGTAACACACGCTATTCACAGATTTTCCAGGGCAGAGTCACCATGACCAGGG
ACACATCCGCGAGCACAGCCTACATGGAGCTGAGCAGCCTGAGATCTGAAGACA
CGGCTGTGTATTACTGTGCGAGAAACAAGCCTGGAATGTGGGAGGTTGACTACT
GGGGCCAAGGAACCCTGGTCACCGTCTCCTCA

QVQLVQSGAEVKKPGASVKVSCKASGYTFTSYAMHWVRQAPGQRPEWMGWISVD
NGNTRYSQIFQGRVTMTRDTSASTAYMELSSLRSEDTAVYYCARNKPGMWEVDYW
GQGTLVTVSS

>P4V6-13 VL
GACATCGTGATGACCCAGTCTCCAGACTCCCTGGCTGTGTCTCTGGGCGAGAGGG
CCACCATCAACTGCAAGTCCAGCCAGAGTGTTTTATACAGCTCCAACAATAAGA
ACTACTTAGCTTGGTACCAGCAGAAACCAGGACAGCCTCCTAAGCTGCTCATTTA
CTGGGCATCTACCCGGGAATCCGGGGTCCCTGACCGATTCAGTGGCAGCGGGTCT
GGGACAGATTTCACTCTCACCATCAGCAGCCTGCAGGCTGAAGATGTGGCAGTTT
ATTACTGTCAGCAGTATAATACCTGGCCTCGGCTCACTTTCGGCCAGGGGACCAA
GCTGGAGATCAAA

DIVMTQSPDSLAVSLGERATINCKSSQSVLYSSNNKNYLAWYQQKPGQPPKLLIYWA
STRESGVPDRFSGSGSGTDFTLTISSLQAEDVAVYYCQQYNTWPRLTFGQGTKLEIK

>E1-HC
CAGGTGCAGCTGGTGCAGTCTGGGGCTGAGGTGAAGAAGCCTGGGGCCTCAGTG
AAGGTTTCCTGCAAGGCATCTGGATACACCTTCACCAGCTACTATATGCACTGGG
TGCGACAGGCCCCTGGACAAGGGCTTGAGTGGATGGGAATAATCAACCCTAGTG

GTGGTAGCACAAGCTACGCACAGAAGTTCCAGGGCAGAGTCACCATGACCAGGG
ACACGTCCACGAGCACAGTCTACATGGAGCTGAGCAGCCTGAGATCTGAGGACA
CGGCCGTGTATTACTGTGCGAGAGAATACACAGGGTCCAAGGCTTTTGACTACTG
GGGCCAAGGAACCCTGGTCACCGTCTCCTCA

QVQLVQSGAEVKKPGASVKVSCKASGYTFTSYYMHWVRQAPGQGLEWMGIINPSG
GSTSYAQKFQGRVTMTRDTSTSTVYMELSSLRSEDTAVYYCAREYTGSKAFDYWG
QGTLVTVSS

>E1-LC
GATATTGTGATGACCCAGACTCCACTCTCTCTGTCCGTCACCCCTGGACAGCCGG
CCTCCATCTCCTGCAAGTCTAGTCAGAGCCTCCTGCATAGTGATGGAAAGACCTA
TTTGTATTGGTACCTGCAGAAGCCAGGCCAGTCTCCACAGCTCCTAATCTATGAA
GTTTCCAGCCGGTTCTCTGGAGTGCCAGATAGGTTCAGTGGCAGCGGGTCAGGG
ACAGATTTCACACTGAAAATCAGCCGGGTGGAGGCTGAGGATGTTGGGGTTTATT
ACTGCATGCAAGATGGACACCTTCCTCGGACTTTCGGCGGAGGGACCAAGGTGG
AGATCAAA

DIVMTQTPLSLSVTPGQPASISCKSSQSLLHSDGKTYLYWYLQKPGQSPQLLIYEVSS
RFSGVPDRFSGSGSGTDFTLKISRVEAEDVGVYYCMQDGHLPRTFGGGTKVEIK

>E2-HC
CAGGTGCAGCTGGTGCAGTCTGGGGCTGAGGTGAAGAAGCCTGGGGCCTCAGTG
AAGGTTTCCTGCAAGGCATCTGGATACACCTTCACCAGCTACTATATGCACTGGG
TGCGACAGGCCCCTGGACAAGGGCTTGAGTGGATGGGAATAATCAACCCTAGTG
GTGGTAGCACAAGCTACGCACAGAAGTTCCAGGGCAGAGTCACCATGACCAGGG
ACACGTCCACGAGCACAGTCTACATGGAGCTGAGCAGCCTGAGATCTGAGGACA
CGGCCGTGTATTACTGTGCGAGAGACAGTGGGAGCTACTCCCCTGACTACTGGG
GCCAAGGAACCCTGGTCACCGTCTCCTCA

QVQLVQSGAEVKKPGASVKVSCKASGYTFTSYYMHWVRQAPGQGLEWMGIINPSG
GSTSYAQKFQGRVTMTRDTSTSTVYMELSSLRSEDTAVYYCARDSGSYSPDYWGQG
TLVTVSS

>E2-LC
CAGACTGTGGTGACTCAGGAGCCATCGTTCTCAGTGTCCCCTGGAGGGACAGTCA
CACTCACTTGTGGCTTGAGCTCTGGCTCAGTCTCTACTAGTTACTACCCCAGCTGG
TACCAGCAGACCCCAGGCCAGGCTCCACGCACGCTCATCTACAGCACAAACACT
CGCTCTTCTGGGGTCCCTGATCGCTTCTCTGGCTCCATCCTTGGGAACAAAGCTG
CCCTCACCATCACGGGGGCCCAGGCAGATGATGAATCTGATTATTACTGTGTGCT
GTATATGGGTAATGGCATTTATGTCTTCGGAACTGGGACCAAGGTCACCGTCCTA

QTVVTQEPSFSVSPGGTVTLTCGLSSGSVSTSYYPSWYQQTPGQAPRTLIYSTNTRSS
GVPDRFSGSILGNKAALTITGAQADDESDYYCVLYMGNGIYVFGTGTKVTVL

>E3-HC
CAGGTGCAGCTGGTGCAGTCTGGGGCTGAGGTGAAGAAGCCTGGGGCCTCAGTG
AAGGTTTCCTGCAAGGCATCTGGATACACCTTCACCAGCTACTATATGCACTGGG
TGCGACAGGCCCCTGGACAAGGGCTTGAGTGGATGGGAATAATCAACCCTAGTG
GTGGTAGCACAAGCACGCACAGAAGTTCCAGGGCAGAGTCACCATGACCAGGGA
CACGTCCACGAGCACAGTCTACATGGAGCTGAGCAGCCTGAGATCTGAGGACAC
GGCCGTGTATTACTGTGCGACAGGATGGCCACGATCTCTTGACTACTGGGGCCAA
GGAACCCTGGTCACCGTCTCCTCA

QVQLVQSGAEVKKPGASVKVSCKASGYTFTSYYMHWVRQAPGQGLEWMGIINPSG
GSTSYAQKFQGRVTMTRDTSTSTVYMELSSLRSEDTAVYYCATGWPRSLDYWGQG
TLVTVSS

>E3-LC
CAGTCTGTGCTGACTCAGCCACCCTCAGCGTCTGGGACCCCCGGGCAGAGGGTC
ACCATCTCTTGTTCTGGAAGCAGCTCCAACATCGGAAGTAATACTGTAAACTGGT
ACCAGCAGCTCCCAGGAACGGCCCCCAAACTCCTCATCTATAGTAATAATCAGC
GGCCCTCAGGGGTCCCTGACCGATTCTCTGGCTCCAAGTCTGGCACCTCAGCCTC
CCTGGCCATCAGTGGGCTCCAGTCTGAGGATGAGGCTGATTATTACTGTGCAATG
TGGGATGACAGCCTGGATGGTCCGGTGTTCGGCGGAGGGACCAAGCTGACCGTC
CTAG

QSVLTQPPSASGTPGQRVTISCSGSSSNIGSNTVNWYQQLPGTAPKLLIYSNNQRPSG
VPDRFSGSKSGTSASLAISGLQSEDEADYYCAMWDDSLDGPVFGGGTKLTVL

>E4-HC
CAGGTGCAGCTGGTGCAGTCTGGGGCTGAGGTGAAGAAGCCTGGGGCCTCAGTG
AAGGTCTCCTGCAAGGCTTCTGGATACACCTTCACCGGCTACTATATGCACTGGG
TGCGACAGGCCCCTGGACAAGGGCTTGAGTGGATGGGACGGATCAACCCTAACA
GTGGTGGCACAAACTATGCACAGAAGTTTCAGGGCAGGGTCACCAGTACCAGGG
ACACGTCCATCAGCACAGCCTACATGGAGCTGAGCAGGCTGAGATCTGACGACA
CGGTCGTGTATTACTGTGGGGCAGAACGGACCTTTTTTTATAGTTCGGGGAGTTC
GGCGAGTTATTATAACGAGGGGAATGCTTTTGATGTCTGGGGCCAAGGGACAAT
GGTCACCGTCTCTTCA

QVQLVQSGAEVKKPGASVKVSCKASGYTFTGYYMHWVRQAPGQGLEWMGRINPN
SGGTNYAQKFQGRVTSTRDTSISTAYMELSRLRSDDTVVYYCGAERTFFYSSGSSAS
YYNEGNAFDVWGQGTMVTVSS

>E4-LC
CAGTCTGTGCTGACTCAGCCACCCTCAGCGTCTGGGACCCCCGGGCAGAGGGTC
ACCATCTCTTGTTCTGGAAGCAGCTCCAACATCGGAAGTAATTATGTATACTGGT
ACCAGCAGCTCCCAGGAACGGCCCCCAAACTCCTCATCTATAGGAATAATCAGC
GGCCCTCAGGGGTCCCTGACCGATTCTCTGGCTCCAAGTCTGGCACCTCAGCCTC
CCTGGCCATCAGTGGGCTCCGGTCCGAGGATGAGGCTGATTATTACTGTGCATCT
TGGGATGACGGCCTGAGTGGTCGGATGTTCGGCGGAGGGACCAAGCTGACCGTC
CTA

QSVLTQPPSASGTPGQRVTISCSGSSSNIGSNYVYWYQQLPGTAPKLLIYRNNQRPSG
VPDRFSGSKSGTSASLAISGLRSEDEADYYCASWDDGLSGRMFGGGTKLTVL

>E5-HC
CAGGTGCAGCTGGTGCAGTCTGGGGCTGAGGTGAAGAAGCCTGGGTCCTCGGTG
AAGGTCTCCTGCAAGGCTTCTGGAGGCACCTTCAGCAGCTATGCTATCAGCTGGG
TGCGACAGGCCCCTGGACAAGGGCTTGAGTGGATGGGAGGGATCATCCCTATCT
TTGGTACAGCAAACTACGCACAGAAGTTCCAGGGCAGAGTCACGATTACCGCGG
ACGAATCCACGAGCACAGCCTACATGGAGCTGAGCAGCCTGAGATCTGAGGACA
CGGCCGTGTATTACTGTACGAAAATGGGTGGGACCTACTCTTTTGACTCCTGGGG
CCAAGGAACCCTGGTCACCGTCTCCTCA

QVQLVQSGAEVKKPGSSVKVSCKASGGTFSSYAISWVRQAPGQGLEWMGGIIPIFGT
ANYAQKFQGRVTITADESTSTAYMELSSLRSEDTAVYYCTKMGGTYSFDSWGQGTL
VTVSS

>E5-LC
TCCTATGTGCTGACTCAGCCACCCTCGGTGTCAGTGGCCCCAGGACAGACGGCCA
GGATTACCTGTGGGGGAAACAACATTGGAAGTAAAAGTGTGCACTGGTACCAGC
AGAAGCCAGGCCAGGCCCCTGTGCTGGTCGTCTATGATGATAGCGACCGGCCCT
CAGGGATCCCTGAGCGATTCTCTGGCTCCAACTCTGGGAACACGGCCACCCTGAC
CATCAGCAGGGTCGAAGCCGGGGATGAGGCCGACTATTACTGTCAGGTGTGGGA
TCGTACTACTGATCAAGTGGTGTTCGGCGGAGGGACCAAGCTGACCGTCCTA

SYVLTQPPSVSVAPGQTARITCGGNNIGSKSVHWYQQKPGQAPVLVVYDDSDRPSGI
PERFSGSNSGNTATLTISRVEAGDEADYYCQVWDRTTDQVVFGGGTKLTVL

>E6-HC
CAGGTGCAGCTGGTGCAGTCTGGGGCTGAGGTGAAGAAGCCTGGGTCCTCGGTG
AAGGTCTCCTGCAAGGCTTCTGGAGGCACCTTCAGCAGCTATGCTATCAGCTGGG
TGCGACAGGCCCCTGGACAAGGGCTTGAGTGGATGGGAGGGATCATCCCTATCT
TTGGTACAGCAAACTACGCACAGAAGTTCCAGGGCAGAGTCACGATTACCGCGG

ACGAATCCACGAGCACAGCCTACATGGAGCTGAGCAGCCTGAGATCTGAGGACA
CGGCCGTGTATTACTGTGCGAGAGTTTTGCGTCCTGGGCTACAACGGGGTGGTT
CGACACCTGGGGCCAAGGAACCCTGGTCACCGTCTCCTCA

QVQLVQSGAEVKKPGSSVKVSCKASGGTFSSYAISWVRQAPGQGLEWMGGIIPIFGT
ANYAQKFQGRVTITADESTSTAYMELSSLRSEDTAVYYCARVFASWATTGWFDTW
GQGTLVTVSS

>E6-LC
GACATCCAGATGACCCAGTCTCCATCCTCCCTGTCTGCATCTGTAGGAGACAGAG
TCACCATCACTTGCCGGGCGAGTCAGGGCATTAGCAATTATTTAGCCTGGTATCA
GCAGAAACCAGGGAAAGTTCCTAAGCTCCTGATCTATGCTGCATCCACTTTGCAA
TCAGGGGTCCCATCTCGGTTCAGTGGCAGTGGATCTGGGACAGATTTCACTCTCA
CCATCAGCAGCCTGCAGCCTGAAGATGTTGCAACTTATTACTGTCAAAAGTATGA
CAGTGCCCCGCCCACTTTCGGCGGAGGGACCAAGGTGGAGATCAAA

DIQMTQSPSSLSASVGDRVTITCRASQGISNYLAWYQQKPGKVPKLLIYAASTLQSGV
PSRFSGSGSGTDFTLTISSLQPEDVATYYCQKYDSAPPTFGGGTKVEIK

>G1-HC
CAGGTCCAGCTTGTGCAGTCTGGGGCTGAGGTGAAGAAGCCTGGGGCCTCAGTG
AAGGTTTCCTGCAAGGCTTCTGGATACACCTTCACTAGCTATGCTATGCATTGGG
TGCGCCAGGCCCCCGGACAAAGGCTTGAGTGGATGGGATGGATCAACGCTGGCA
ATGGTAACACAAAATATTCACAGAAGTTCCAGGGCAGAGTCACCATTACCAGGG
ACACATCCGCGAGCACAGCCTACATGGAGCTGAGCAGCCTGAGATCTGAAGACA
CGGCTGTGTATTACTGTGCGAGAAACAAGCCTGGAATGTGGGAGGTTGACTACT
GGGGCCAAGGAACCCTGGTCACCGTCTCCTCA

QVQLVQSGAEVKKPGASVKVSCKASGYTFTSYAMHWVRQAPGQRLEWMGWINAG
NGNTKYSQKFQGRVTITRDTSASTAYMELSSLRSEDTAVYYCARNKPGMWEVDYW
GQGTLVTVSS

>G1-LC
GAAATTGTGTTGACGCAGTCTCCAGGCACCCTGTCTTTGTCTCCAGGGGAAAGAG
CCACCCTCTCCTGCAGGGCCAGTCAGAGTGTTAGCAGCAGCTACTTAGCCTGGTA
CCAGCAGAAACCTGGCCAGGCTCCCAGGCTCCTCATCTATGGTGCATCCAGCAG
GGCCACTGGCATCCCAGACAGGTTCAGTGGCAGTGGGTCTGGGACAGACTTCAC
TCTCACCATCAGCAGACTGGAGCCTGAAGATTTTGCAGTGTATTACTGTCAGCAG
TATGGTAGCTCACCCGAGACGTTCGGCCAAGGGACCAAGGTGGAAATCAAA

EIVLTQSPGTLSLSPGERATLSCRASQSVSSSYLAWYQQKPGQAPRLLIYGASSRATGI
PDRFSGSGSGTDFTLTISRLEPEDFAVYYCQQYGSSPETFGQGTKVEIK

>G2-HC
CAGGTGCAGCTGGTGCAGTCTGGGGCTGAGGTGAAGAAGCCTGGGGCCTCAGTG
AAGGTTTCCTGCAAGGCATCTGGATACACCTTCACCAGCTACTATATGCACTGGG
TGCGACAGGCCCCTGGACAAGGGCTTGAGTGGATGGGAATAATCAACCCTAGTG
GTGGTAGCACAAGCTACGCACAGAAGTTCCAGGGCAGAGTCACCATGACCAGGG
ACACGTCCACGAGCACAGTCTACATGGAGCTGAGCAGCCTGAGATCTGAGGACA
CGGCCGTGTATTACTGTGTGAGAGTCCTGCCGGGGTATAACCTGCTGTGGGGATT
TGACTATTGGGGCCAAGGAACCCTGGTCACCGTCTCCTCA

QVQLVQSGAEVKKPGASVKVSCKASGYTFTSYYMHWVRQAPGQGLEWMGIINPSG
GSTSYAQKFQGRVTMTRDTSTSTVYMELSSLRSEDTAVYYCVRVLPGYNLLWGFDY
WGQGTLVTVSS

>G2-LC
GATaTTGTGATGACTCAGTCTCCACTCTCCCTGCCCGTCACCCCTGGAGAGCCGGC
CTCCATCTCCTGCAGGTCTAGTCAGAGCCTCCTCCATAGTAATGGATACAACTAT
TTGGATTGGTACCTGCAGAAGCCAGGGCAGTCTCCACAGCTCCTGATCTATTTGG
GTTCTAATCGGGCCTCCGGGGTCCCTGACAGGTTCAGTGGCAGTGGATCAGGCAC
AGATTTTACACTGAAAATCAGCAGAGTGGAGGCTGAGGATGTTGGGGTTTATTAC
TGCATGCAAACTCTACAAAGTCCTCCCACTTTCGGCCCTGGGACCAAAGTGGATA
TCAAA

DVVMTQSPLSLPVTPGEPASISCRSSQSLLHSNGYNYLDWYLQKPGQSPQLLIYLGSN
RASGVPDRFSGSGSGTDFTLKISRVEAEDVGVYYCMQTLQSPPTFGPGTKVDIK

>G3-HC
GAGGTGCAGCTGGTGGAGTCCGGGGGAGGCTTAGTTCAGCCTGGGGGGTCCCTG
AGACTCTCCTGTGCAGCCTCTGGATTCACCTTCAGTAGCTACTGGATGCACTGGG
TCCGCCAAGCTCCAGGGAAGGGGCTGGTGTGGGTCTCACGTATTAATAGTGATG
GGAGTAGCACAAGCTACGCGGACTCCGTGAAGGGCCGATTCACCATCTCCAGAG
ACAACGCCAAGAACACGCTGTATCTGCAAATGAACAGTCTGAGAGCCGAGGACA
CGGCTGTGTATTACTGTGGAAGAGATTATTATAGCACTGTGGACTACTGGGGCCA
AGGAACCCTGGTCACCGTCTCCTCA

EVQLVESGGGLVQPGGSLRLSCAASGFTFSSYWMHWVRQAPGKGLVWVSRINSDG
SSTSYADSVKGRFTISRDNAKNTLYLQMNSLRAEDTAVYYCGRDYYSTVDYWGQG
TLVTVSS

>G3-LC
CAGTCTGCCCTGACTCAGCCTGCCTCCGTGTCTGGGTCTCCTGGACAGTCGATCA
CCATCTCCTGCACTGGAACCAGCAGTGACGTTGGTGGTTATAACTATGTCTCCTG

GTACCAACAGCACCCAGGCAAAGCCCCCAAACTCATGATTTATGAGGTCAGTAA
TCGGCCCTCAGGGGTTTCTAATCGCTTCTCTGGCTCCAAGTCTGGCAACACGGCC
TCCCTGACCATCTCTGGGCTCCAGGCTGAGGACGAGGCTGATTATTACTGCAGCT
CATATACAAGCAGCAACACTGTGCTATTCGGCGGAGGGACCAAGCTGACCGTCC
TA

QSALTQPASVSGSPGQSITISCTGTSSDVGGYNYVSWYQQHPGKAPKLMIYEVSNRPS
GVSNRFSGSKSGNTASLTISGLQAEDEADYYCSSYTSSNTVLFGGGTKLTVL

>G4-HC
GAGGTGCAGCTGGTGGAGTCTGGGGGAGGCTTGGTCCAGCCTGGAGGGTCCCTG
AGACTCTCCTGTGCAGCCTCTGGATTCACCTTCAGTGACCACTACATGGACTGGG
TCCGCCAGGCTCCAGGGAAGGGGCTGGAGTGGGTTGGCCGTACTAGAAACAAAG
CTAACAGTTACACCACAGAATACGCCGCGTCTGTGAAAGGCAGATTCACCATCTC
AAGAGATGATTCAAAGAACTCACTGTATCTGCAAATGAACAGCCTGAAAACCGA
GGACACGGCCGTGTATTACTGTACCACTTATTGGGGGAGCCGCACTCCCTTTGAC
AACTGGGGCCAAGGAACCCTGGTCACCGTCTCCTCA

EVQLVESGGGLVQPGGSLRLSCAASGFTFSDHYMDWVRQAPGKGLEWVGRTRNKA
NSYTTEYAASVKGRFTISRDDSKNSLYLQMNSLKTEDTAVYYCTTYWGSRTPFDNW
GQGTLVTVSS

>G4-LC
GACATCCAGATGACCCAGTCTCCATCCTCCCTGTCTGCATCTGTAGGAGACAGAG
TCACCATCACTTGCCGGGCAAGTCAGGGCATTAGAAATGATTTAGGCTGGTATCA
GCAGAAACCAGGGAAAGCCCCTAAGCGCCTGATCTATGCTGCATCCAGTTTGCA
AAGTGGGGTCCCATCAAGGTTCAGCGGCAGTGGATCTGGGACAGAATTCACTCT
CACAATCAGCAGCCTGCAGCCTGAAGATTTTGCAACTTATTACTGTCTACAGCAT
CAGAGTTACCCACTCACTTTCGGCGGAGGGACCAAGGTGGAGATCAAA

DIQMTQSPSSLSASVGDRVTITCRASQGIRNDLGWYQQKPGKAPKRLIYAASSLQSG
VPSRFSGSGSGTEFTLTISSLQPEDFATYYCLQHQSYPLTFGGGTKVEIK

>G5-HC
CAGGTGCAGCTGGTGGAGTCTGGGGGAGGCGTGGTCCAGCCTGGGAGGTCCCTG
AGACTCTCCTGTGCAGCCTCTGGATTCACCTTCAGTAGCTATGCTATGCACTGGG
TCCGCCAGGCTCCAGGCAAGGGGCTAGAGTGGGTGGCAGTTATATCATATGATG
GAAGTAATAAATACTACGCAGACTCCGTGAAGGGCCGATTCACCATCTCCAGAG
ACAATTCCAAGAACACGCTGTATCTGCAAATGAACAGCCTGAGAGCTGAGGACA
CGGCTGTGTATTACTGTGCGAAAGGGTCTGCAGCAGCTGCAGTCCTTGACGGCTG
GGGCCAAGGAACCCTGGTCACCGTCTCCTCA

QVQLVESGGGVVQPGRSLRLSCAASGFTFSSYAMHWVRQAPGKGLEWVAVISYDG
SNKYYADSVKGRFTISRDNSKNTLYLQMNSLRAEDTAVYYCAKGSAAAAVLDGWG
QGTLVTVSS

>G5-LC
GACATCGTGATGACCCAGTCTCCAGACTCCCTGGCTGTGTCTCTGGGCGAGAGGG
CCACCATCAACTGCAAGTCCAGCCAGAGTGTTTTATACAGCTCCAACAATAAGA
ACTACTTAGCTTGGTACCAGCAGAAACCAGGACAGCCTCCTAAGCTGCTCATTTA
CTGGGCATCTACCCGGGAATCCGGGGTCCCTGACCGATTCAGTGGCAGCGGGTCT
GGGACAGATTTCACTCTCACCATCAGCAGCCTGCAGGCTGAAGATGTGGCAGTTT
ATTACTGTCAGCAATATTATAGAACTCCCCTCACTTTCGGCGGAGGGACCAAGGT
GGAGATCAAA

DIVMTQSPDSLAVSLGERATINCKSSQSVLYSSNNKNYLAWYQQKPGQPPKLLIYWA
STRESGVPDRFSGSGSGTDFTLTISSLQAEDVAVYYCQQYYRTPLTFGGGTKVEIK

>G6-HC
CAGGTGCAGCTACAGCAGTGGGGCGCAGGACTGTTGAAGCCTTCGGAGACCCTG
TCCCTCACCTGCGCTGTCTATGGTGGGTCCTTCAGTGGTTACTACTGGAGCTGGA
TCCGCCAGCCCCCAGGGAAGGGGCTGGAGTGGATTGGGGAAATCAATCATAGTG
GAAGCACCAACTACAACCCGTCCCTCAAGAGTCGAGTCACCATATCAGTAGACA
CGTCCAAGAACCAGTTCTCCCTGAAGCTGAGCTCTGTGACCGCCGCGGACACGG
CTGTGTATTACTGTGCGAGAGAAAATGAACCTAGTGGTTATGACTATTGGGGCCA
AGGAACCCTGGTCACCGTCTCCTCA

QVQLQQWGAGLLKPSETLSLTCAVYGGSFSGYYWSWIRQPPGKGLEWIGEINHSGS
TNYNPSLKSRVTISVDTSKNQFSLKLSSVTAADTAVYYCARENEPSGYDYWGQGTL
VTVSS

>G6-LC
GACATCGTGATGACCCAGTCTCCACTCTCCCTGCCCGTCACCCCTGGAGAGCCGG
CCTCCATCTCCTGCAGGTCTAGTCAGAGCCTCCTGCATAGTAATGGATACAACTA
TTTGGATTGGTACCTGCAGAAGCCAGGGCAGTCTCCACAGCTCCTGATCTATTTG
GGTTCTAATCGGGCCTCCGGGGTCCCTGACAGGTTCAGTGGCAGTGGATCAGGC
ACAGATTTTACACTGAAAATCAGCAGAGTGGAGGCTGAGGATGTTGGGGTTTATT
ACTGCATGCAAGCTCTACAAACTCCCACTTTCGGCGGAGGGACCAAGGTGGAGA
TCAAA

DIVMTQSPLSLPVTPGEPASISCRSSQSLLHSNGYNYLDWYLQKPGQSPQLLIYLGSN
RASGVPDRFSGSGSGTDFTLKISRVEAEDVGVYYCMQALQTPTFGGGTKVEIK

Supplementary Dataset S2: Sequences of Spike-RBD(aa319-591)-mFC

ATGACCCGGCTGACAGTCCTGGCCCTGCTGGCTGGTCTGCTGGCGTCCTCTAGAG
CCAGAGTCCAACCAACAGAATCTATTGTTAGATTTCCTAATATTACAAACTTGTG
CCCTTTTGGTGAAGTTTTTAACGCCACCAGATTTGCATCTGTTTATGCTTGGAACA
GGAAGAGAATCAGCAACTGTGTTGCTGATTATTCTGTCCTATATAATTCCGCATC
ATTTTCCACTTTTAAGTGTTATGGAGTGTCTCCTACTAAATTAAATGATCTCTGCT
TTACTAATGTCTATGCAGATTCATTTGTAATTAGAGGTGATGAAGTCAGACAAAT
CGCTCCAGGGCAAACTGGAAAGATTGCTGATTATAATTATAAATTACCAGATGAT
TTTACAGGCTGCGTTATAGCTTGGAATTCTAACAATCTTGATTCTAAGGTTGGTG
GTAATTATAATTACCTGTATAGATTGTTTAGGAAGTCTAATCTCAAACCTTTTGAG
AGAGATATTTCAACTGAAATCTATCAGGCCGGTAGCACACCTTGTAATGGTGTTG
AAGGTTTTAATTGTTACTTTCCTTTACAATCATATGGTTTCCAACCCACTAATGGT
GTTGGTTACCAACCATACAGAGTAGTAGTACTTTCTTTTGAACTTCTACATGCAC
CAGCAACTGTTTGTGGACCTAAAAAGTCTACTAATTTGGTTAAAAACAAATGTGT
CAATTTCAACTTCAATGGTTTAACAGGCACAGGTGTTCTTACTGAGTCTAACAAA
AAGTTTCTGCCTTTCCAACAATTTGGCAGAGACATTGCTGACACTACTGATGCTG
TCCGTGATCCACAGACACTTGAGATTCTTGACATTACACCATGTTCTGGAGGCGG
CGGCTCCGAGCCCAGAGGGCCCACAATCAAGCCCTGTCCTCCATGCAAATGCCC
AGCACCTAACCTCTTGGGTGGACCATCCGTCTTCATCTTCCCTCCAAAGATCAAG
GATGTACTCATGATCTCCCTGAGCCCCATAGTCACATGTGTGGTGGTGGATGTGA
GCGAGGATGACCCAGATGTCCAGATCAGCTGGTTTGTGAACAACGTGGAAGTAC
ACACAGCTCAGACACAAACCCATAGAGAGGATTACAACAGTACTCTCCGGGTGG
TCAGTGCCCTCCCCATCCAGCACCAGGACTGGATGAGTGGCAAGGAGTTCAAAT
GCAAGGTCAACAACAAAGACCTCCCAGCGCCCATCGAGAGAACCATCTCAAAAC
CCAAAGGGTCAGTAAGAGCTCCACAGGTATATGTCTTGCCTCCACCAGAAGAAG
AGATGACTAAGAAACAGGTCACTCTGACCTGCATGGTCACAGACTTCATGCCTG
AAGACATTTACGTGGAGTGGACCAACAACGGGAAAACAGAGCTAAACTACAAG
AACACTGAACCAGTCCTGGACTCTGATGGTTCTTACTTCATGTACAGCAAGCTGA
GAGTGGAAAAGAAGAACTGGGTGGAAAGAAATAGCTACTCCTGTTCAGTGGTCC
ACGAGGGTCTGCACAATCACCACACGACTAAGAGCTTCTCCCGGACTCCGGGTA
AATAA

MTRLTVLALLAGLLASSRA**RVQPTESIVRFPNITNLCPFGEVFNATRFASVYAWNR**
KRISNCVADYSVLYNSASFSTFKCYGVSPTKLNDLCFTNVYADSFVIRGDEVRQI
APGQTGKIADYNYKLPDDFTGCVIAWNSNNLDSKVGGNYNYLYRLFRKSNLKP
FERDISTEIYQAGSTPCNGVEGFNCYFPLQSYGFQPTNGVGYQPYRVVVLSFELL
HAPATVCGPKKSTNLVKNKCVNFNFNGLTGTGVLTESNKKFLPFQQFGRDIAD
TTDAVRDPQTLEILDITPCSGGGGSEPRGPTIKPCPPCKCPAPNLLGGPSVFIFPPKIK
DVLMISLSPIVTCVVVDVSEDDPDVQISWFVNNVEVHTAQTQTHREDYNSTLRVVSA
LPIQHQDWMSGKEFKCKVNNKDLPAPIERTISKPKGSVRAPQVYVLPPPEEEMTKKQ

VTLTCMVTDFMPEDIYVEWTNNGKTELNYKNTEPVLDSDGSYFMYSKLRVEKKNW
VERNSYSCSVVHEGLHNHHTTKSFSRTPGK
